# Efficient production of large-size optical Schrödinger cat states

**DOI:** 10.1038/s41598-019-50703-1

**Published:** 2019-10-04

**Authors:** Evgeny V. Mikheev, Alexander S. Pugin, Dmitry A. Kuts, Sergey A. Podoshvedov, Nguyen Ba An

**Affiliations:** 1 Laboratory of Quantum Information Processing and Quantum Computing, Institute of Natural and Exact Sciences, South Ural State University (SUSU), Lenin Av. 76, Chelyabinsk, Russia; 2 Department of Applied Mathematics and Programming, Institute of Natural and Exact Sciences, South Ural State University (SUSU), Lenin Av. 76, Chelyabinsk, Russia; 3 Thang Long Institute of Mathematics and Applied Sciences (TIMAS), Thang Long University (TLU), Nghiem Xuan Yem, Hoang Mai, Hanoi Vietnam; 4 Institute of Physics, Vietnam Academy of Science and Technology (VAST), 18 Hoang Quoc Viet, Cau Giay, Hanoi Vietnam

**Keywords:** Qubits, Theoretical physics

## Abstract

We present novel theory of effective realization of large-size optical Schrödinger cat states, which play an important role for quantum communication and quantum computation in the optical domain using laser sources. The treatment is based on the *α*-representation in infinite Hilbert space which is the decomposition of an arbitrary quantum state in terms of displaced number states characterized by the displacement amplitude *α*. We find analytical form of the *α*-representation for both even and odd Schrödinger cat states which is essential for their generation schemes. Two schemes are proposed for generating even/odd Schrödinger cat states of large size |*β*| (|*β*| ≥ 2) with high fidelity *F* (*F* ≈ 0.99). One scheme relies on an initially offline prepared two-mode entangled state with a fixed total photon number, while the other scheme uses separable photon Fock states as the input. In both schemes, generation of the desired states is heralded by the corresponding measurement outcomes. Conditions for obtaining states useful for quantum information processing are established and success probabilities for their generation are evaluated.

## Introduction

It is known that a potentially quantum computer can effectively implement intractable algorithms such as large integer factoring^1^ and unsorted data search^2^ which cannot be effectively implemented by computers operating under classical laws. But realization of the quantum computer requires effective performance of a universal set of deterministic gate operations over a large set of qubits^3^. Also, qubits are exposed to influence of the environment, requiring good fault-tolerant computational systems. All these impose highly stringent requirements on the physical system where qubits and quantum gates are realized. Different physical systems might be used to implement different quantum protocols. In particular, as light has the maximally possible speed of propagation and weakly interacts with the surrounding noisy environment, optical systems are put in one row with atomic ones in the design of possible configurations of the quantum computer.

Although there are many proposed approaches for optical quantum computers, none of them are completely satisfactory since they are quite complex and/or restricted in application. For example, realization of deterministic gate operations^4^ would require an unacceptably huge number of additional operations^5,6^. So, one can hardly say that the issue of optical quantum information processing (QIP) has been finally resolved^7^ and the question of how to efficiently exploit the optical resources (interaction mechanisms, approaches, suitable states) for QIP remains of great interest. Up to now, three approaches for optical QIP are developed within the discrete-variable (DV)^5^, continuous-variable (CV)^8^ and combined discrete-continuous-variable (DV-CV) frameworks^9^. These approaches exploit one of aspects of the particle-wave duality^7^ or both of them^10,11^. Each approach has its own inherent advantages and drawbacks. Namely, the DV approach uses photons that interact very weakly with each other so two-qubit operations can be realized only in a non-deterministic manner^12^. Instead, quantum protocols with CV states can be implemented deterministically, but the fidelity is limited due to the fact that CV entangled states such as two-mode squeezed vacuum state does not carry maximum entanglement^13^. Commonly used optical states are the so-called optical Schrödinger cat states (SCSs) ${a}_{0}|-\alpha \rangle \pm {a}_{1}|\alpha \rangle$, with |±*α*〉 being coherent states with macroscopic continuous amplitudes ±*α* and *a*_0_, *a*_1_ normalization coefficients. These states can also be referred to as quantum superpositions of two out-of-phase light pulses. The size of coherent components |*α*| is of crucial importance in the experiments to test quantum foundations and quantum information technologies^14–18^. Generally |−*α*〉 and |*α*〉 are not strictly orthogonal to each other. But, since their overlap is determined by $|\langle \alpha |-\alpha \rangle |=exp(-2|\alpha {|}^{2})$, for $|\alpha |\ge 2$ one has $|\langle \alpha |-\alpha \rangle |\le 3\cdot {10}^{-4}\approx 0$, so such SCSs can be treated as good qubits. They are called large-size SCSs where “large-size” practically implies |*α*| ≥ 2. However, it is very difficult to produce such large-size SCSs in realistic conditions with existing third-order nonlinearities *χ*^(3)^. Although a lot of progress has been made over last times^19–26^, size of the generated SCSs as well as their low generation rate still leave much to be improved for desired practical protocols. In other words, the realization of sufficiently large-size SCSs remains questionable and is worth further tremendous efforts. In this connection, the DV-CV approach with the so-called hybrid states turns out to be a promising direction since the combination of two different physical systems could provide new capabilities to more efficiently implement optical quantum protocols^27–36^.

Since the direct implementation of the SCSs^37^ is currently impossible due to the tininess of the third-order optical nonlinearity, it makes sense to consider other methods^38^ that could approximate large-size SCSs with high fidelity. A scheme for generating SCSs by feeding a squeezed vacuum into beam splitter and counting photons in auxiliary mode was considered in^39^. It was also shown^40^ that any single-mode quantum state can be generated from the vacuum by alternate applications of displacement operations combined with single photons. For the time being, the techniques of photon subtraction and photon addition are fairly common for generating different types of SCSs^17,21,25,26,41–43^. These techniques are widely demonstrated in modern optical experiments^44–50^. Recently, a seemingly simple method^51^ has been proposed. The method seems simple because it needs only a balanced beam-splitter and a quadrature measurement, provided that the proper initial states are supplied beforehand. However, preparation of the proper initial states, which are themselves SCSs, though, of small-size, is not trivial. In fact, they have first to prepare squeezed vacuum states and then subtract photon to obtain the necessary initial small SCSs, whose fidelity due to the technique is not so high (just 84% as reported in the reference).

Here, we present novel ways to directly generate even/odd SCSs of large size without the breeding process as in the above-mentioned method^51^ that could be directly used in work of quantum computer. The method is based on introduction of the so-called *α*-representation of even/odd SCSs which is their decomposition in base of the displaced number states^17^. One method is based on the pre-preparation of a two-mode entangled state with a fixed number *n* of photons^50^. Photon subtraction from the displaced number state^52,53^ of the original entangled one in auxiliary mode allows one to generate the states that under certain conditions approximate either even or odd large-size SCSs with fidelity close to or even more of 0.99 that are suitable for quantum protocols. This approach allows one to find strategy for generating auxiliary two-mode entangled states taking into account experimental conditions and imperfections imposed in reality. The method can be considered efficient because the necessary auxiliary two-mode entangled states^50^ needs just to be prepared offline in advance. We also develop another method for conditional generation of even/odd large-size SCSs by mixing photon Fock states on beam splitters followed by displacing the auxiliary modes and subsequently measuring their photon numbers by photo-detectors. Both the proposed schemes allow us to effectively generate even/odd SCSs with large size and with high fidelity.

## Results

### Schrödinger cat states in *iα*-representation

The even/odd SCSs $|{\beta }_{\pm }\rangle$ with size |*β*| are defined by

1 $$|{\beta }_{+}\rangle ={N}_{+}(|-\beta \rangle +|\beta \rangle ),$$

2 $$|{\beta }_{-}\rangle ={N}_{-}(|-\beta \rangle -|\beta \rangle ),$$

where ${N}_{\pm }={(2(1\pm exp(-2{|\beta |}^{2})))}^{-1/2}$ are the normalization factors, which in general depend on |*β*| and the notations $|\pm \beta \rangle$ mean coherent states with amplitudes ±*β*. The amplitude *β* is generally complex, but here and in the following, for simplicity, it is assumed to be real and positive (i.e., $\beta  > 0)$. Then the amplitude *β* of the SCS is regarded as its size.

The even/odd SCSs are obviously orthogonal to each other, $\langle {\beta }_{-}|{\beta }_{+}\rangle =0,$ as the photon numbers in $|{\beta }_{+}\rangle$ ($|{\beta }_{-}\rangle$) are even (odd). In this paper we are working with the so-called *iα*-representation which for any state is determined in infinite Hilbert space of the displaced number states (S1) of Supplementary Note 1 characterized by the displacement amplitude *α* (see refs^17,18^). Precisely, the *iα*-representation of an arbitrary state is its decomposition over the basis states $\{|k,i\alpha \rangle ;\,k=0,1,\ldots ,\infty \}$ of the displaced number states. In the case of the optical SCSs, we have (see Supplementary Note 1 accompanying this Main Material)

3 $$|{\beta }_{+}\rangle ={N}_{+}exp(-\frac{{{\mathbb{a}}}^{2}}{2}){\sum }_{k=0}^{\infty }{a}_{k}^{(+)}|k,i\alpha \rangle ,$$

4 $$|{\beta }_{-}\rangle ={N}_{-}exp(-\frac{{{\mathbb{a}}}^{2}}{2}){\sum }_{k=0}^{\infty }{a}_{k}^{(-)}|k,{\rm{i}}\alpha \rangle ,$$

where the decomposition coefficients ${a}_{k}^{(\pm )}$ for a given *β* read

5 $${a}_{k}^{(+)}=\frac{2{(i{\mathbb{a}})}^{k}}{\sqrt{k!}}cos(\alpha \beta +k(\phi +\pi /2)),$$

6 $${a}_{k}^{(-)}=\frac{2{(i{\mathbb{a}})}^{k}}{\sqrt{k!}}sin(\alpha \beta +k(\phi +\pi /2)),$$

with *α* being real while the relative phase $\phi =arctang(\alpha /\beta )$ and ${\mathbb{a}}=\sqrt{{|\alpha |}^{2}+{|\beta |}^{2}}$. The choice of real *α* (i.e., the displacement amplitude *iα* in (3) and (4) is purely imaginary) looks convenient since then the value of the parameter*iα* will lie symmetrically with respect to the quantities −*β* and *β* on the phase plane. It is possible to directly check that the normalization condition is satisfied for both even and odd SCSs, i.e., ${N}_{\pm }^{2}exp(-{{\mathbb{a}}}^{2}){\sum }_{n=0}^{\infty }{|{a}_{n}^{(\pm )}|}^{2}=1$ hold for any values of the parameters *α* and *β*. We can see that the coefficients may not be equal to zero for arbitrary values of *k*. We obtain standard form of the coefficients of the even/odd SCSs in the Fock or number state basis (or, the same, in the 0-representation) if we take *α* = 0 in Eqs (5, 6). The division into ‘even’ and ‘odd’ takes place exclusively in the 0-representation of the SCSs. In any *α*-representations with $\alpha \ne 0$, the division into ‘even’ and ‘odd’ is not relevant, because, as seen from Eqs (5, 6), they contain both even and odd displaced states. Nevertheless, we still formally adopt the terminologies ‘even’ and ‘odd’ SCSs even in α-presentations with $\alpha \ne 0$ that should not cause a conceptual misleading.

### Schrödinger cat qudits

It is long known that the size of SCSs generated by direct use of *χ*^(3)^ nonlinearities^14^ cannot be large enough due to the tininess of the nonlinearities available in all existing nonlinear crystals. Quantum engineering allows the replacement of the original infinite CV state with its finite version which represents a truncated superposition of just *n* + 1 terms in the corresponding *α*-representation, with *n* being some integer. That is, we can approximate the SCSs in Eqs (1, 2) by the following states

7 $$|{\Psi }_{n}^{(\pm )}\rangle ={N}_{n}^{(\pm )}{\sum }_{k=0}^{n}{b}_{k}^{(\pm )}|k,i\alpha \rangle ,$$

with ${b}_{k}^{(\pm )}$ some expansion coefficients to be specified later and ${N}_{n}^{(\pm )}={({\sum }_{k=0}^{n}{|{b}_{k}^{(\pm )}|}^{2})}^{-1/2}$ the normalization factors. We can also speak about replacing original optical SCSs in Eqs (1, 2), which are CV states residing in an infinite Hilbert space, by the states in Eq. (7), which are DV states residing in a finite Hilbert space of dimension *d* = *n* + 1. The degree of validity for such a replacement can be assessed by the fidelity ${F}_{n}^{(\pm )}=tr({{\varrho }}_{n}^{(\pm )}{{\varrho }}^{(SCS)}),$ with *tr* denoting the trace over the state in parentheses, ${{\varrho }}^{(SCS)}$ is the density matrix of the original pure states in Eqs (3, 4) and ${{\varrho }}_{n}^{(\pm )}$ is the density matrix of the states in Eq. (7). The fidelity value lies in the range from 0 up to 1. If the fidelity is equal to 1, then the compared states are identical to each other. Conversely, if the fidelity is equal to 0, then such states are orthogonal to each other. The bigger value the fidelity acquires the closer to each other are the two compared states. In the case of the optical SCSs in Eqs (3, 4) and their truncated versions in Eq. (7), the fidelity can be written as

8 $${F}_{n}^{(+)}={|\langle {\beta }_{+}{|\Psi }_{n}^{(+)}\rangle |}^{2}={N}_{+}^{2}{N}_{n}^{(+)2}exp(-{{\mathbb{a}}}^{2}){|{\sum }_{k=0}^{n}{a}_{k}^{{(+)}^{\ast }}{b}_{k}^{(+)}|}^{2},$$

9 $${F}_{n}^{(-)}={|\langle {\beta }_{-}{|\Psi }_{n}^{(-)}\rangle |}^{2}={N}_{-}^{2}{N}_{n}^{(-)2}exp(-{{\mathbb{a}}}^{2}){|{\sum }_{k=0}^{n}{a}_{k}^{{(-)}^{\ast }}{b}_{k}^{(-)}|}^{2}.$$

By numerical calculations we find out that the best way to approximate the original SCSs in Eqs (1, 2) with highest fidelity is to set the expansion coefficients in Eq. (7) to be proportional to those in Eqs (5, 6), say, in the following way: ${b}_{k}^{(+)}={a}_{k}^{(+)}/2$ and ${b}_{k}^{(-)}={a}_{k}^{(-)}/2$. Let us denote the states in Eq. (7) with such setting for the coefficients by $|{\Psi }_{n}^{(S+)}\rangle$ and loosely call them Schrödinger cat qudits (SCQs) of dimension *d* = *n* + 1 which have the following form

10 $$\begin{array}{c}|{\Psi }_{n}^{(S+)}\rangle ={N}_{n}^{(S+)}{\sum }_{k=0}^{n}({a}_{k}^{(+)}/2)|k,i\alpha \rangle =\\ {N}_{n}^{(S+)}\mathop{\sum }\limits_{k=0}^{n}({(i{\mathbb{a}})}^{k}/\sqrt{k!})cos(\alpha \beta +k(\phi +\pi /2))|k,i\alpha \rangle \end{array}$$

and

11 $$|{\Psi }_{n}^{(S-)}\rangle ={N}_{n}^{(S+)}{\sum }_{k=0}^{n}({a}_{k}^{(-)}/2)|k,i\alpha \rangle ={N}_{n}^{(S-)}\mathop{\sum }\limits_{k=0}^{n}\,({(i{\mathbb{a}})}^{k}/\sqrt{k!})sin(\alpha \beta +k(\phi +\pi /2))|k,i\alpha \rangle ,$$

with the corresponding normalization factors

12 $${N}_{n}^{(S+)}={({\sum }_{k=0}^{n}({{\mathbb{a}}}^{2k}/k!)co{s}^{2}(\alpha \beta +k(\phi +\pi /2)))}^{-1/2},$$

13 $${N}_{n}^{(S-)}={({\sum }_{k=0}^{n}({{\mathbb{a}}}^{2k}/k!)si{n}^{2}(\alpha \beta +k(\phi +\pi /2)))}^{-1/2},$$

by virtue of Eqs (10, 11). Then, we can derive from Eqs (8, 9) the expressions for the fidelities ${F}_{n}^{({\rm{S}}\pm )}$ between the original SCSs in Eqs (3, 4) and the approximated ones, i.e., the SCQs in Eqs (10, 11):

14 $${F}_{n}^{({\rm{S}}\pm )}=({N}_{\pm }^{2}{N}_{n}^{(S\pm )2}exp(\,-\,{{\mathbb{a}}}^{2})/4){\sum }_{k=0}^{n}{|{a}_{k}^{(\pm )}|}^{2}.$$

The functions ${F}_{n}^{({\rm{S}}\pm )}$ depend not only on *n* but also on two independent variables *α* and *β*. The generic tendency is that ${F}_{n}^{(S\pm )}$ increase and approach 1 for increasing *n*, while the range of the values of *α* and *β*, in which high fidelities are achieved, also increases with *n*. All the material relating to the properties of the SCQs is presented in the accompanying Supplementary Notes. This material is the basis for the manipulation and generation of the even/odd SCQs which will be described in what follows.

**Figure 1:**
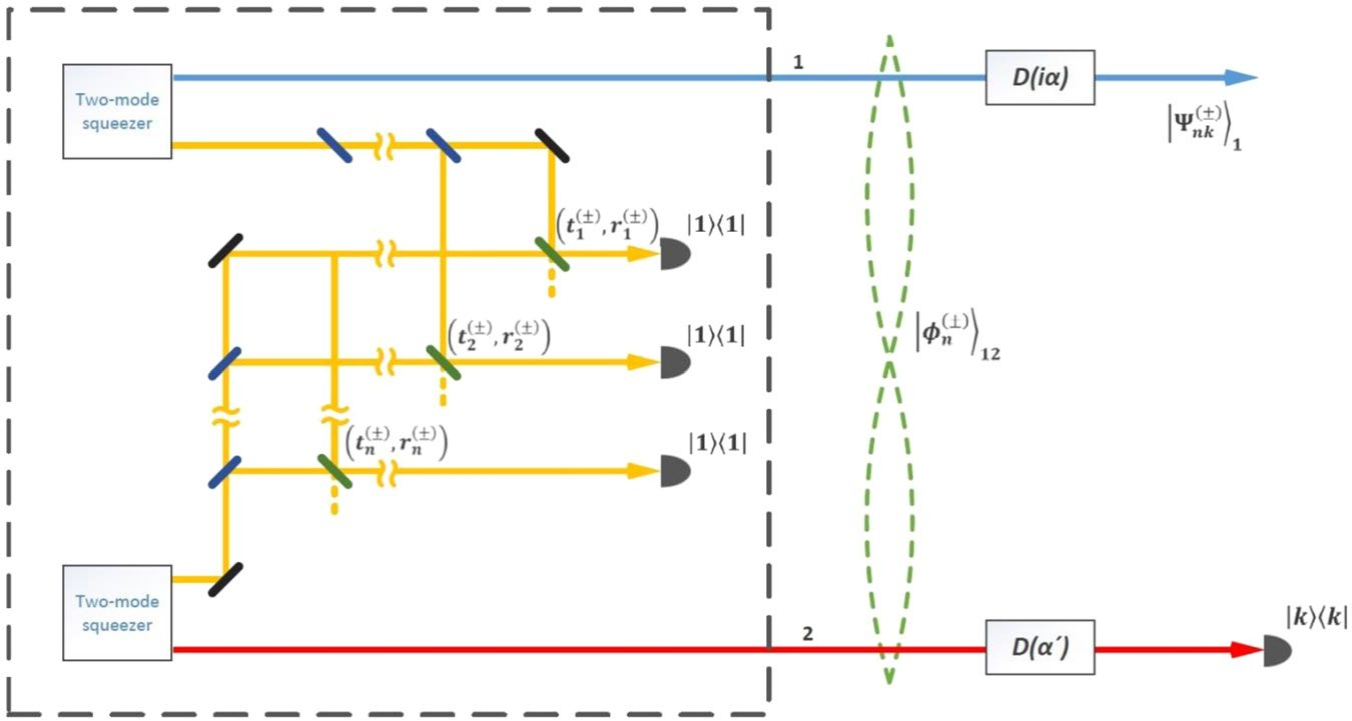
Schematic representation for generation of the SCQs in Eqs (10, 11) together with the auxiliary part (inside a rectangle surrounded by a dashed line) responsible for generating the two-mode entangled state in Eq. (15). Two HTBS are used to displace initially prepared entangled two-mode states ${|{\varphi }_{n}^{(\pm )}\rangle }_{12}$ of Eq. (15) by quantities *iα* and *α*′, respectively. Conditioned on registration of *k* photons in mode 2, the initial state ${|{\varphi }_{n}^{(\pm )}\rangle }_{12}$ is projected onto ${|{\Psi }_{nk}^{(\pm )}\rangle }_{1}$ which may approximate even/odd optical SCSs. The auxiliary part consists of two coupled two-mode squeezers idler modes of which are converted in a rather complicated way by a system of the beam splitter with parameters $({t}_{i}^{(\pm )},{r}_{i}^{(\pm )})$ and mirrors^50^. Desired quantum superposition in Eq. (15) occupying signal modes is generated in a heralded fashion provided that the parameters $({t}_{i}^{(\pm )},{r}_{i}^{(\pm )})$ are selected as indicated in the text.

**Figure 2:**
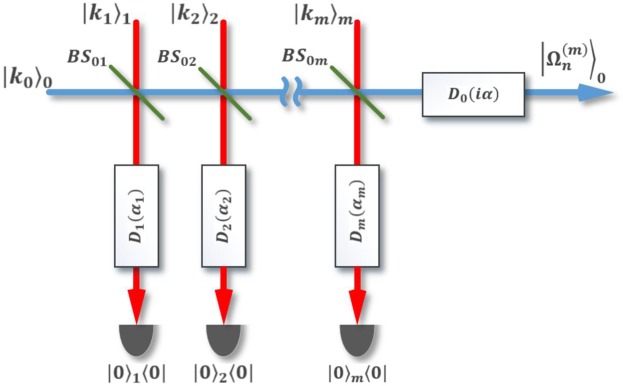
Schematic setup for generation of state $|{\Omega }_{n}^{(m)}\rangle$ in Eq. (34) starting from Fock states. $|{k}_{j}{\rangle }_{j}$ denotes an input Fock state containing *k*_*j*_ photons in mode *j*, $B{S}_{0j}$ beam splitter with transmission (reflection) coefficient ${t}_{j}\,({r}_{j})$ acting on mode 0 and mode *j*, ${D}_{j}(\alpha )\,$ displacement operator with displace amplitude *α* acting on mode *j*, $|0{\rangle }_{j}\langle 0|$ implies detection of no photons in mode *j*, and $|{\Omega }_{n}^{(m)}$ the output post-selected state.

 We also note the fact that, in general, we can consider the SCQ in an arbitrary *α*-representation, where the magnitude *α* can take arbitrary complex value $\alpha =Re\alpha +iIm\alpha$. The choice of a purely imaginary value *iα* with real *α* is deliberate (in addition to the above-mentioned fact that the value of *iα* lies on the imaginary axis on phase plane, which is the axis of symmetry for real values *β* and −*β*), since the numerical simulation shows that the fidelities of the SCQ in Supplementary Figures 1–3 of Supplementary Note 1 are maximum with *iα* compared to arbitrary *α*. For this reason, the final displacement operator with purely imaginary displacement amplitude *iα* will be used in the optical schemes in the Figs 1 and 2. The choice of a purely imaginary value *iα* imposes certain rules on the SCQ’s amplitudes. For example, as it follows from formulas (5) and (6), the imaginary unit alternates depending on the number of the term in the superposition, the odd terms are purely imaginary and the even members are real. This circumstance imposes an appropriate choice on auxiliary states in Figs 1 and 2 to provide this alternation of the imaginary unit in superposition terms for the construction of the SCQs.

### Schemes for generation of SCQ

#### Scheme using a two-mode entangled state as the input

The SCQs in Eqs (10, 11) that approximate the desired SCSs in Eqs (1, 2) with high fidelity can be generated by our scheme shown in Fig. 1, exploiting the following two-mode entangled state

15 $${|{\varphi }_{n}^{(\pm )}\rangle }_{12}={\sum }_{m=0}^{n}{d}_{m}^{(\pm )}|m{\rangle }_{1}|n-m{\rangle }_{2},$$

with the coefficients ${d}_{m}^{(\pm )}$ satisfying the normalization conditions ${\sum }_{m=0}^{n}{|{d}_{m}^{(\pm )}|}^{2}=1,$ as the initial state. Note that in Eq. (15) the photon number of either mode may be any between 0 and *n* but the total photon number of two modes is fixed to *n*. Given the coefficients ${d}_{m}^{(\pm )},$ the state in Eq. (15) can be pre-produced offline in a conditional optical setup with two spontaneous parametric down converters (SPDCs) connected with each other by a set of properly-arranged beam splitters^50^ (see more later). Note that the optical scheme in Fig. 1 includes both the scheme for generating SCQ and the preliminary part for producing the necessary two-mode entangled state of *n* photons. The part of the multi-stage scheme that is responsible for generating the state is placed inside a dashed rectangle. In Fig. 1 mode 1 is the main, where the SCQ is to be born, while mode 2 is the auxiliary one, whose photon number is to be detected (|*kk*| implies that *k* photons are registered by a detector).

Starting from the state ${|{\varphi }_{n}^{(\pm )}\rangle }_{12}$ two displacement operators *D*_1_(*iα*) and *D*_2_(*α*′) (*α* ≠ *α*′ in general) act respectively on mode 1 and mode 2, resulting in

16 $${D}_{1}(i\alpha ){D}_{2}(\alpha ^{\prime} ){|{\varphi }_{n}^{(\pm )}\rangle }_{12}={\sum }_{m=0}^{n}{d}_{m}^{(\pm )}{|m,i\alpha \rangle }_{1}{|n-m,\alpha ^{\prime} \rangle }_{2},$$

where $|m,i\alpha \rangle ={D}_{1}(i\alpha )|m{\rangle }_{1}$ and $|n-m,\alpha ^{\prime} \rangle ={D}_{2}(\alpha ^{\prime} )|n-m,\alpha ^{\prime} {\rangle }_{2}$ are the displaced number states. It is worth noting that the displacement operation can be realized by mixing the target state with a strong coherent state on a highly transmissive beam splitter (HTBS)^54,55^. Then, measurement on the auxiliary mode 2 in Fig. 1 is carried out in the number states basis {|*k*〉; *k* = 0, 1, 2, …}. Using the decomposition of the displaced number state over number states as in Eq. (S5) of Supplementary Note 1, the state (16) can be reformulated as

17 $${D}_{1}(i\alpha ){D}_{2}(\alpha ^{\prime} ){|{\varphi }_{n}^{(\pm )}\rangle }_{12}=F(\alpha ^{\prime} ){\sum }_{k=0}^{\infty }{N}_{nk}^{(\pm )-1}{|{\Psi }_{nk}^{(\pm )}\rangle }_{1}|k{\rangle }_{2},$$

where the state of mode 1

18 $${|{\Psi }_{nk}^{(\pm )}\rangle }_{1}={N}_{nk}^{(\pm )}{\sum }_{m=0}^{n}{d}_{m}^{(\pm )}{c}_{n-m,k}(\alpha ^{\prime} )|m,i\alpha {\rangle }_{1}$$

is normalized with the normalization factor

19 $${N}_{nk}^{(\pm )}={({\sum }_{m=0}^{n}{|{d}_{m}^{(\pm )}|}^{2}{|{c}_{n-m,k}(\alpha ^{\prime} )|}^{2})}^{-1/2}.$$

As seen from Eq. (17), conditioned on the outcome *k* of the measurement on mode 2 (i.e., mode 2 is found in state $|{k}_{2}\rangle$ or, the same, *k* photons of mode 2 are detected), mode 1 is immediately projected onto the state ${|{\Psi }_{nk}^{(\pm )}\rangle }_{1}$ of Eq. (18). Note that in Eq. (18) the subscripts ‘*nk*’ imply generation of a qudit of dimension *n* + 1 in mode 1 which is heralded by detection of *k* photons in mode 2, while superscripts ‘±’ refer to even/odd SCQs. The exponential multiplier *F*(*α*)′ in in Eq. (17) is introduced in Supplementary Note 1. The success probability to generate the state in Eq. (18) is determined by

20 $${P}_{nk}^{(\pm )}={F}^{2}(\alpha ^{\prime} ){N}_{nk}^{(\pm )-2}=exp(\,-\,{|\alpha ^{\prime} |}^{2})(\mathop{\sum }\limits_{m=0}^{n}{|{d}_{m}^{(\pm )}|}^{2}{|{c}_{n-m,k}(\alpha ^{\prime} )|}^{2}).$$

Using the completeness of the displaced number states, it is straightforward to check that all the success probabilities sum to one, i.e.,

21 $${\sum }_{k=0}^{\infty }{P}_{nk}^{(\pm )}=1,$$

for any value of *α*′ and *n*, as it should be.

Furthermore, if we impose conditions on the coefficients ${d}_{m}^{(\pm )}$ of the initial state in Eq. (15) as

22 $${d}_{m}^{(+)}=\frac{{a}_{m}^{(+)}/2}{{c}_{n-m,k}(\alpha ^{\prime} )}{N}_{nk}^{(S+)^{\prime} }=\frac{{(i{\rm{a}})}^{m}cos(\alpha \beta +m(\phi +\pi /2))}{{c}_{n-m,k}(\alpha ^{\prime} )\sqrt{m!}}{N}_{nk}^{(S+)^{\prime} },$$

or

23 $${d}_{m}^{(-)}=\frac{{a}_{m}^{(-)}/2}{{c}_{n-m,k}(\alpha ^{\prime} )}{N}_{nk}^{(S-)^{\prime} }=\frac{{(i{\rm{a}})}^{m}sin(\alpha \beta +m(\phi +\pi /2))}{{c}_{n-m,k}(\alpha ^{\prime} )\sqrt{m!}\,}{N}_{nk}^{(S-)^{\prime} }$$

with the normalization factors

24 $${N}_{nk}^{(S\pm )^{\prime} }={(\sqrt{{\sum }_{m=0}^{n}{|{a}_{m}^{(\pm )}|}^{2}/4{|{c}_{n-m,k}(\alpha ^{\prime} )|}^{2}})}^{-1},$$

we shall obtain the desired SCQs in Eqs (10) and (11) whose fidelities are plotted in Supplementary Figures 1 and 2, respectively. The expressions (24) for the factors ${N}_{nk}^{(S\pm )^{\prime} }$, which are present in the coefficients ${d}_{m}^{(\pm )}$ in Eqs (22, 23), ensure the normalization of the generated SCQs. Here, we wish to note a fact that the interaction of the modes of the initial state in Eq. (15) with the coherent state on the HTBS leaves its imprint in the form of the coefficients ${c}_{n-m,k}(\alpha ^{\prime} )$ in the generated SCQs (i.e., the state $|{\Psi }_{nk}^{(\pm )}\rangle$ in Eq. (18)). It can serve inherent irreducible feature of the DV-CV interaction. The success probabilities to generate the SCQs in Eqs (10, 11) are given by

25 $${P}_{nk}^{(S\pm )}={F}^{2}{N}_{nk}^{(S\pm )^{\prime} 2}/{N}_{n}^{(S\pm )2}$$

whose dependences on the involved parameters are plotted and displayed in Supplementary Figures 5–8 of Supplementary Note 2).

At this point, we briefly address on a possibility to generate the two-mode entangled state ${|{\varphi }_{n}^{(\pm )}\rangle }_{12}$ in Eq. (15), following the work of ref.^50^. For concreteness, let us reformulate the state in Eq. (15) in terms of the bosonic modal creation operators ${a}_{1}^{+}$ and ${a}_{2}^{+}$ as

26 $${|{\varphi }_{n}^{(+)}\rangle }_{12}={\sum }_{m=0}^{n}\frac{{d}_{m}^{(\pm )}}{\sqrt{m!(n-m)!}}{a}_{1}^{+m}{a}_{2}^{+(n-m)}|0{\rangle }_{1}|0{\rangle }_{2},$$

with $|{0}_{1}\rangle |{0}_{2}\rangle$ the two-mode vacuum state. If we pull ${a}_{2}^{+n}$ out of the sum and introduce a formal variable $z={a}_{1}^{+}/{a}_{2}^{+}$, then Eq. (26) reads

27 $${|{\varphi }_{n}^{(+)}\rangle }_{12}={a}_{2}^{+n}f(z)|0{\rangle }_{1}|0{\rangle }_{2},$$

where

28 $$f(z)={\sum }_{m=0}^{n}\frac{{d}_{m}^{(\pm )}}{\sqrt{m!(n-m)!}}{z}^{m}$$

is a nonconstant single-variable *n*^*th*^ order polynomial in *z* with complex coefficients. According to the fundamental theorem of algebra, the above polynomial *f*(*z*) can always be factorized out as

29 $$f(z)=\frac{{d}_{n}^{(+)}}{\sqrt{n!}}{\prod }_{m=0}^{n}(z-{z}_{m}^{(+)}),$$

with ${z}_{m}^{(+)}$ solutions of the equation *f*(*z*) = 0. Putting Eq. (29) back into Eq. (27) yields

30 $${|{\varphi }_{n}^{(+)}\rangle }_{12}=\frac{{d}_{n}^{(+)}}{\sqrt{n!}}\,[{\prod }_{m=0}^{n}({a}_{1}^{+}-{z}_{m}^{(+)}{a}_{2}^{+})]\,|0{\rangle }_{1}|0{\rangle }_{2}.$$

By changing the variables ${z}_{m}^{(+)}\to -{r}_{m}^{(+)}/{t}_{m}^{(+)}$, with ${r}_{m}^{(+)}$ and ${t}_{m}^{(+)}$ such that $|{r}_{m}^{(+)}{|}^{2}$ + $|{t}_{m}^{(+)}{|}^{2}=1,$ we get

31 $${|{\varphi }_{n}^{(+)}\rangle }_{12}=\frac{{d}_{n}^{(+)}}{\sqrt{n!}{\prod }_{m=0}^{n}{t}_{m}^{(+)}}[{\prod }_{m=0}^{n}({t}_{m}^{(+)}{a}_{1}^{+}+{r}_{m}^{(+)}{a}_{2}^{+})]\,|0{\rangle }_{1}|0{\rangle }_{2}.$$

The parameters ${t}_{m}^{(+)}$ and ${r}_{m}^{(+)}$ can be treated as transmission and reflection coefficients of a beam splitter which are determined by ${z}_{m}^{(+)}$ in the following manner

32 $${t}_{m}^{(+)}=\frac{1}{\sqrt{1+{|{z}_{m}^{(+)}|}^{2}}},$$

33 $${r}_{m}^{(+)}=-\frac{{z}_{m}^{(+)}}{\sqrt{1+{|{z}_{m}^{(+)}|}^{2}}}.$$

Because the state in Eq. (31) is a product of terms that are linear in the modal creation operators acting on the two-mode vacuum state, such states can be generated by a heralded scheme proposed in ref.^50^. We present the scheme in Fig. 1 in conjunction with the main one used for generation of the SCQs. The scheme starts from two two-mode squeezed states produced by two independent SPDCs. Each squeezed state has a signal mode and an idler mode. First, each idler mode is splitted into *n* modes with an equal weight by a set of *n* − 1 unbalanced beam splitters with proper transmission and reflection coefficients. Then, the splitted modes from one idler mode are correspondingly superposed with those from the other idler mode on *n* beam splitters with transmission and reflection coefficients $({t}_{1}^{(+)},{r}_{1}^{(+)}),$ $({t}_{2}^{(+)},{r}_{2}^{(+)}),$ … and $({t}_{n}^{(+)},{r}_{n}^{(+)}),$ respectively. Behind each such beam splitter there is a photo-detector. If each detector registers a photon, then the two signal modes are projected onto the state ${|{\varphi }_{n}^{(+)}\rangle }_{12}$. The same procedures apply to generation of the state ${|{\varphi }_{n}^{(-)}\rangle }_{12}$. The state generation process described above is probabilistic but this does not matter since ${|{\varphi }_{n}^{(\pm )}\rangle }_{12}$ are to be generated offline and only after they are successfully generated we shall turn to the problem of generation of our SCQs as in Fig. 1. Because analytically finding the solutions $\{{z}_{m}^{(\pm )};\,m=0,1,\ldots ,n\}$ for specific coefficients $\{{d}_{m}^{(\pm )};m=0,1,2,\ldots ,n\}$ is generally not easy, we, for illustration, carry out numerical calculation for the cases of *n* = 3, 6, 9 and 12 and some given values of the displacement amplitudes *α* and *α*′ (see Fig. 1) for which the fidelities ${F}_{n}^{({\rm{S}}\pm )} > \mathrm{0.99.}$ The calculated values of ${t}_{m}^{(\pm )}$ and ${r}_{m}^{(\pm )}$ are collected in Tables 1 and 2. As can be seen from the Tables, high-fidelity SCQs of large size ($\beta \ge 2$) can be produced in the case of relatively large *n* (say, *n* ≥ 9). Since the concerned optical devices (SPDCs, beam splitters, phase shifters, …) are available within the current technologies and the necessary numerical calculation is not formidable with the help of modern computing facilities, the presented production of large-size optical Schrödinger cat states seems quite efficient. As can be seen from the tables, the parameters of the beam splitters $({t}_{j}^{(\pm )},{r}_{j}^{(\pm )})$ include, in general, complex values, in particular, to ensure the alternation of imaginary units in superposition terms (Eqs (5) and (6)) of the generated states.

**Table 1:** Values of the beam splitter parameters ${t}_{i}^{(+)}$, ${r}_{i}^{(+)}$ use of which in optical scheme of ref.^50^ ensures the generation of the needed two-mode entangled state in Eq. (15).

	$|{{\boldsymbol{\Psi }}}_{{\bf{3}}}^{({\boldsymbol{S}}{\boldsymbol{+}})}\rangle$	$|{{\boldsymbol{\Psi }}}_{{\bf{6}}}^{({\boldsymbol{S}}{\boldsymbol{+}})}\rangle$	$|{{\boldsymbol{\Psi }}}_{{\bf{9}}}^{({\boldsymbol{S}}{\boldsymbol{+}})}\rangle$	$|{{\boldsymbol{\Psi }}}_{{\bf{12}}}^{({\boldsymbol{S}}{\boldsymbol{+}})}\rangle$
*β*	1.03	1.64	2.12	2.25
*α*	0.328	0	0.23	0
*α*′	1.426	1.805	2.048	2.248
${{\rm{P}}}_{n0}^{(S+)}$	0.20	0.12	0.08	0.06
${{\rm{t}}}_{1}^{(+)}$	0.755	0.582	0.574	0.563
${{\rm{r}}}_{1}^{(+)}$	$-i0.656$	$0.814exp(i0.399\pi )$	$0.818exp(\,-\,i0.299\pi )$	$0.826exp(\,-\,i0.286\pi )$
${{\rm{t}}}_{2}^{(+)}$	0.634	0.582	0.577	0.563
${{\rm{r}}}_{2}^{(+)}$	$i0.773$	$0.814exp(\,-\,i0.399\pi )$	$0.817exp(i0.363\pi )$	$0.826exp(i0.286\pi )$
${{\rm{t}}}_{3}^{(+)}$	0.547	0.883	0.698	0.663
${{\rm{r}}}_{3}^{(+)}$	$-i0.837$	*i*0.469	$0.716exp(\,-\,i0.469\pi )$	$0.749exp(\,-\,i0.428\pi )$
${{\rm{t}}}_{4}^{(+)}$		0.883	0.97	0.663
${{\rm{r}}}_{4}^{(+)}$		$-i0.469$	$-i0.242$	$0.749exp(i0.428\pi )$
${{\rm{t}}}_{5}^{(+)}$		0.582	0.904	0.964
${{\rm{r}}}_{5}^{(+)}$		$0.814exp(\,-\,i0.601\pi )$	*i*0.428	*i*0.264
${{\rm{t}}}_{6}^{(+)}$		0.582	0.674	0.964
${{\rm{r}}}_{6}^{(+)}$		$0.814exp(i0.601\pi )$	*i*0.739	$-i0.264$
${{\rm{t}}}_{7}^{(+)}$			0.698	0.774
${{\rm{r}}}_{7}^{(+)}$			$0.716exp(\,-\,i0.531\pi )$	$-i0.633$
${{\rm{t}}}_{8}^{(+)}$			0.577	0.774
${{\rm{r}}}_{8}^{(+)}$			$0.817exp(i0.637\pi )$	*i*0.633
${{\rm{t}}}_{9}^{(+)}$			0.574	0.663
${{\rm{r}}}_{9}^{(+)}$			$0.818exp(\,-\,i0.701\pi )$	$0.749exp(i0.572\pi )$
${{\rm{t}}}_{10}^{(+)}$				0.663
${{\rm{r}}}_{10}^{(+)}$				$0.749exp(\,-\,i0.572\pi )$
${{\rm{t}}}_{11}^{(+)}$				0.563
${{\rm{r}}}_{11}^{(+)}$				$0.826exp(i0.714\pi )$
${{\rm{t}}}_{12}^{(+)}$				0.563
${{\rm{r}}}_{12}^{(+)}$				$0.826exp(\,-\,i0.714\pi )$

Application of the displacement operators with amplitudes *α* and *α*′ in Fig. 1 enables us to generate SCQ that most closely match the properties of the even SCS of the corresponding size *β* with fidelity ${F}_{n}^{({\rm{S}}+)} > 0.99$.

**Table 2:** Values of the beam splitter parameters ${t}_{i}^{(-)}$, ${r}_{i}^{(-)}$ use of which in optical scheme of ref.^50^ ensures the generation of the needed two-mode entangled state (15).

	$|{{\boldsymbol{\Psi }}}_{{\bf{3}}}^{({\boldsymbol{S}}{\boldsymbol{-}})}\rangle$	$|{{\boldsymbol{\Psi }}}_{{\bf{6}}}^{({\boldsymbol{S}}{\boldsymbol{-}})}\rangle$	$|{{\boldsymbol{\Psi }}}_{{\bf{9}}}^{({\boldsymbol{S}}{\boldsymbol{-}})}\rangle$	$|{{\boldsymbol{\Psi }}}_{{\bf{12}}}^{({\boldsymbol{S}}{\boldsymbol{-}})}\rangle$
*β*	1.04	1.62	2.13	2.54
*α*	0	0.266	0	0.205
*α*′	1.265	1.77	2.042	2.248
${{\rm{P}}}_{n0}^{(S-)}$	0.25	0.13	0.08	0.06
${{\rm{t}}}_{1}^{(-)}$	1	0.575	0.574	0.562
${{\rm{r}}}_{1}^{(-)}$	0	$0.818exp(\,-\,i0.356\pi )$	$0.819exp(i0.331\pi )$	$0.827exp(\,-\,i0.262\pi )$
${{\rm{t}}}_{2}^{(-)}$	0.473	00596	0.574	0.563
${{\rm{r}}}_{2}^{(-)}$	*i*0.881	$0.802exp(i0.449\pi )$	$0.819exp(\,-\,i0.331\pi )$	$0.826exp(i0.311\pi )$
${{\rm{t}}}_{3}^{(-)}$	0.473	0.989	1	0.663
${{\rm{r}}}_{3}^{(-)}$	$-i0.881$	*i*0.149	0	$0.748exp(\,-\,i0.403\pi )$
${{\rm{t}}}_{4}^{(-)}$		0.737	0.81	0.665
${{\rm{r}}}_{4}^{(-)}$		$-i0.676$	*i*0.586	$0.747exp(i0.448\pi )$
${{\rm{t}}}_{5}^{(-)}$		0.596	0.81	0.996
${{\rm{r}}}_{5}^{(-)}$		$0.802exp(i0.551\pi )$	$-i0.586$	*i*0.091
${{\rm{t}}}_{6}^{(-)}$		0.575	0.659	0.909
${{\rm{r}}}_{6}^{(-)}$		$0.818exp(\,-\,i0.644\pi )$	*i*0.752	$-i0.417$
${{\rm{t}}}_{7}^{(-)}$			0.659	0.842
${{\rm{r}}}_{7}^{(-)}$			$-i0.752$	*i*0.540
${{\rm{t}}}_{8}^{(-)}$			0.574	0.724
${{\rm{r}}}_{8}^{(-)}$			$0.819exp(i0.669\pi )$	$-i0.690$
${{\rm{t}}}_{9}^{(-)}$			0.574	0.665
${{\rm{r}}}_{9}^{(-)}$			$0.819exp(\,-\,i0.669\pi )$	$0.747exp(i0.552\pi )$
${{\rm{t}}}_{10}^{(-)}$				0.663
${{\rm{r}}}_{10}^{(-)}$				$0.748exp(\,-\,i0.597\pi )$
${{\rm{t}}}_{11}^{(-)}$				0.563
${{\rm{r}}}_{11}^{(-)}$				$0.826exp(i0.689\pi )$
${{\rm{t}}}_{12}^{(-)}$				0.562
${{\rm{r}}}_{12}^{(-)}$				$0.827exp(\,-\,i0.738\pi )$

Application of the displacement with amplitudes *α* and *α*′ in Fig. 1 enables us to generate SCQ that most closely match the properties of the odd SCS of the corresponding size *β* with fidelity ${F}_{n}^{({\rm{S}}-)} > 0.99$.

In general, it is also possible to calculate the overall probability of generating the desired states, taking into account the reported results^50^. So we have the probability ${P}_{3}=1.08\cdot {10}^{-5}$ to generate even SCQ with *n* = 3. If we want to increase the number of the terms in the generated superposition up to *n* = 5, then the probability becomes ${P}_{5}=7.42\cdot {10}^{-8}$.

#### Scheme using separable Fock states as the input

The SCQs in Eqs (10, 11) that approximate the desired SCSs in Eqs (1, 2) with high fidelity can also be generated by our second scheme shown in Fig. 2, exploiting $m+1\ge 2$ photon Fock states ${|{k}_{0}\rangle }_{0},$ ${|{k}_{1}\rangle }_{1},\ldots ,{|{k}_{m}\rangle }_{m}$ with ${k}_{0},{k}_{1},\ldots ,{k}_{m}\ge 0$, as the inputs. Such scheme is sketched in Fig. 2 which consists of *m* beam splitters, $m+1$ displacement operations and *m* photo-detectors. For any given *m* ≥ 1 if neither of the *m* detectors clicks, the output state of the form

34 $${|{\Omega }_{n}^{(m)}\rangle }_{0}={N}_{n}^{(m)}{D}_{0}(i\alpha ){\prod }_{k=1}^{n}{D}_{0}({\beta }_{k}^{(m)\ast }){a}^{+}{D}_{0}^{\dagger }({\beta }_{k}^{(m)\ast })|0{\rangle }_{0}$$

is generated, where

35 $$n={k}_{0}+{k}_{1}+\ldots +{k}_{m}$$

and $\{{\beta }_{k}^{(m)};k=1,\,2,\,\ldots ,\,n\}$ being functions of the parameters ${t}_{1},\,{r}_{1}$, $\,{t}_{2},\,{r}_{2}$, …,, $\,{t}_{m},\,{r}_{m}$ of the beam splitters and *α*_1_, *α*_2_, …, *α*_*m*_ of the displacement operators. The state $|{\Omega }_{n}^{(m)}\rangle$ can be made coincident with the desired SCQs $|{\Psi }_{n}^{(S\pm )}\rangle$ by properly choosing the involved parameters. Namely, since the SCQs of Eqs (10, 11) can be rewritten as

36 $$|{\Psi }_{n}^{(S\pm )}\rangle ={\rm{D}}(i\alpha ){\sum }_{k=0}^{n}{c}_{k}^{(S\pm )}|k\rangle ,$$

with ${c}_{k}^{(S\pm )}={N}_{n}^{(S\pm )}{a}_{k}^{(\pm )}/2,$ it can also be expressed in the form (34), i.e.,

37 $$|{\Psi }_{n}^{(S\pm )}\rangle =D(i\alpha )\frac{{c}_{n}^{(S\pm )}}{\sqrt{n!}}{\prod }_{k=1}^{n}D({\gamma }_{k}^{(\pm )\ast }){a}^{+}{D}^{+}({\gamma }_{k}^{(\pm )\ast })|0\rangle ,$$

where {${\gamma }_{k}^{(\pm )};k=1,\,2,\ldots ,n\}$ are the *n* roots of the polynomial

38 $${\sum }_{k=0}^{n}\frac{{c}_{k}^{(S\pm )}\sqrt{n!}}{{c}_{n}^{(S\pm )}\sqrt{k!}}{\gamma }^{(\pm )k}=0.$$

Note that each root ${\gamma }_{k}^{(\pm )}$ depends on the coefficients $\{{c}_{j}^{(S\pm )};j=0,1,\ldots ,n$} of the desired SCQ (36). It follows from comparing states in Eqs (34) and (37) that, for a given set of $\{{c}_{j}^{(S\pm )}\}$, the scheme’s parameters ${t}_{1},\,{r}_{1}$, ${\alpha }_{1}\,{t}_{2},\,{r}_{2}$, *α*_2_…,,$\,{t}_{m},\,{r}_{m},{\alpha }_{m}$ can be chosen such that to satisfy the equations

39 $${N}_{n}^{(m)}=\frac{{c}_{n}^{(S\pm )}}{\sqrt{n!}}$$

and

40 $${\beta }_{k}^{(m)}={\gamma }_{k}^{(\pm )}\,\forall k.$$

If so, $|{\Omega }_{n}^{(m)}\rangle$ becomes $|{\Psi }_{n}^{(S\pm )}\rangle$, implying generation of the desired SCQ from Fock states $|{k}_{0}{\rangle }_{0},$ $|{k}_{1}{\rangle }_{1},\ldots ,|{k}_{m}{\rangle }_{m}$ by our second scheme sketched in Fig. 2.

For illustration, for the $m=1$ case our calculations yield (see Supplementary Note 3)

41 $${N}_{n}^{(1)}=\frac{1}{\sqrt{{P}_{n}^{(1)}}}\frac{{({t}_{1})}^{{k}_{0}}{(-{r}_{1}^{\ast })}^{{k}_{1}}}{\sqrt{{k}_{0}!{k}_{1}!}}exp(-\frac{{|{\alpha }_{1}|}^{2}}{2}),$$

where $n={k}_{0}+{k}_{1}$, ${P}_{n}^{(1)}$ the success probability and

42 $${\beta }_{k}^{(1)}=\{\begin{array}{c}\frac{{r}_{1}}{{t}_{1}}{\alpha }_{1}^{\ast };\,k\in [1,{k}_{0}]\\ -\frac{{t}_{1}^{\ast }}{{r}_{1}^{\ast }}{\alpha }_{1}^{\ast };\,k\in [{k}_{0}+1,{k}_{0}+{k}_{1}]\end{array}.$$

As for the *m* = 2 case we arrive at (see Supplementary Note 3)

43 $${N}_{n}^{(2)}=\frac{1}{\sqrt{{P}_{n}^{(2)}}}\frac{{({t}_{1}{t}_{2})}^{{k}_{0}}{(-{r}_{1}^{\ast }{t}_{2})}^{{k}_{1}}{(-{r}_{2}^{\ast })}^{{k}_{2}}}{\sqrt{{k}_{0}!{k}_{1}!{k}_{2}!}}exp(-\frac{{|{\alpha }_{1}|}^{2}+{|{\alpha }_{2}|}^{2}}{2}),$$

where $n={k}_{0}+{k}_{1}+{k}_{2}$, ${P}_{n}^{(2)}$ the success probability and

44 $${\beta }_{k}^{(2)}=\{\begin{array}{c}\frac{{t}_{1}{r}_{2}{\alpha }_{2}^{\ast }+{r}_{1}{\alpha }_{1}^{\ast }}{{t}_{1}{t}_{2}};\,k\in [1,{k}_{0}]\\ \frac{{r}_{1}^{\ast }{r}_{2}{\alpha }_{2}^{\ast }-{t}_{1}^{\ast }{\alpha }_{1}^{\ast }}{{r}_{1}^{\ast }{t}_{2}};\,k\in [{k}_{0}+1,{k}_{0}+{k}_{1}]\\ \frac{{t}_{2}^{\ast }{\alpha }_{2}^{\ast }}{-{r}_{2}^{\ast }};\,k\in [{k}_{0}+{k}_{1}+1,{k}_{0}+{k}_{1}+{k}_{2}]\end{array}.$$

From the above description, we see that this scheme works for any *m* ≥ 1. It seems that the smaller value of *m* (i.e., the lesser the number of used beam splitters/displacement operators/detectors) the better the scheme with respect to the devices consumption. However, for a given *n*, a smaller value of *m* should be accompanied by larger values of ${k}_{0},{k}_{1},\ldots ,{k}_{m}$ to meet the requirement in Eq. (35). Also, the described scheme is probabilistic because of its post-selection procedure. In fact, there may be a wide range of choice of possible parameters that satisfy the Eqs (39) and (40) with high accuracy; yet each choice leads to a different success probability.

In what follows, for concreteness, let us deal with generation of the SCQs $|{\Psi }_{10}^{(S\pm )}\rangle$ of size *β* = 2 for three sets of (*m*, *k*_0_, *k*_1_, …, *k*_*m*_):

(i) *m* = 3, *k*_0_ = 4, *k*_1_ = *k*_2_ = *k*_3_ = 2,
(ii) *m* = 4, *k*_0_ = 2, *k*_1_ = *k*_2_ = *k*_3_ = *k*_4_ = 2,
(iii) *m* = 5, *k*_0_ = 0, *k*_1_ = *k*_2_ = *k*_3_ = *k*_4_ = *k*_5_ = 2.

The results of numerical calculations are listed in Tables 3–5, respectively.

**Table 3:** Numerical results of the chosen parameters for generation of the SCQs $|{\Psi }_{10}^{(S\pm )}\rangle$ with size *β* = 2 for the case (i), i.e., when $m=3,\,{k}_{0}=4,\,{k}_{1}=\,{k}_{2}={k}_{3}=2.$ ${F}_{10}^{(3)}$ and ${P}_{10}^{(3)}$ are the corresponding fidelity and success probability.

	$|{{\boldsymbol{\Psi }}}_{{\bf{10}}}^{({\boldsymbol{S}}{\boldsymbol{+}})}\rangle$	$|{{\boldsymbol{\Psi }}}_{{\bf{10}}}^{({\boldsymbol{S}}{\boldsymbol{-}})}\rangle$
${F}_{10}^{(3)}$	0.98	0.961
*α*	−0.35	0.44
${P}_{10}^{(3)}$	0.0015	0.0071
*α*_1_	$1.657\cdot exp(i0.485\pi )$	$1.999\cdot exp(i0.161\pi )$
*α*_2_	$0.274\cdot exp(i0.475\pi )$	−0.270
*α*_3_	$1.176\cdot exp(i0.876\pi )$	$1.164\cdot exp(i0.784\pi )$
*t*_1_	$0.614\cdot exp(i0.01\pi )$	$0.732\cdot exp(i0.161\pi )$
*t*_2_	$0.684\cdot exp(i0.6\pi )$	$0.760\cdot exp(i1.216\pi )$
*t*_3_	$0.664\cdot exp(i1.376\pi )$	$0.690\cdot exp(i1.284\pi )$

**Table 4:** Numerical results of the chosen parameters for generation of the SCQs $|{\Psi }_{10}^{(S\pm )}\rangle$ with size *β* = 2 for the case (ii), i.e., when $m=4,\,{k}_{0}=2,\,{k}_{1}=\,{k}_{2}={k}_{3}={k}_{4}=2.$ ${F}_{10}^{(4)}$ and ${P}_{10}^{(4)}$ are the corresponding fidelity and success probability.

	$|{{\boldsymbol{\Psi }}}_{{\bf{10}}}^{({\boldsymbol{S}}{\boldsymbol{+}})}\rangle$	$|{{\boldsymbol{\Psi }}}_{{\bf{10}}}^{({\boldsymbol{S}}{\boldsymbol{-}})}\rangle$
${F}_{10}^{(4)}$	0.985	0.972
*α*	−0.47	−0.14
${P}_{10}^{(4)}$	0.0007	0.0017
*α*_1_	$1.214\cdot exp(-i0.46\pi )$	$1.405\cdot exp(i0.258\pi )$
*α*_2_	$0.841\cdot exp(i0.175\pi )$	$1.026\cdot exp(-i0.905\pi )$
*α*_3_	0	$0.091\cdot exp(i0.223\pi )$
*α*_4_	$1.222\cdot exp(i0.946\pi )$	$1.204\cdot exp(i0.543\pi )$
*t*_1_	$0.755\cdot exp(i0.683\pi )$	$0.603\cdot exp(i0.371\pi )$
*t*_2_	$0.798\cdot exp(i0.056\pi )$	$0.847\cdot exp(i0.158\pi )$
*t*_3_	$0.531\cdot exp(i1.935\pi )$	$0.595\cdot exp(i1.314\pi )$
*t*_4_	$0.829\cdot exp(i0.446\pi )$	0.917

**Table 5:** Numerical results of the chosen parameters for generation of the SCQs $|{\Psi }_{10}^{(S\pm )}\rangle$ with size *β* = 2 for the case (iii), i.e., when $m=5,\,{k}_{0}=0,\,{k}_{1}=\,{k}_{2}={k}_{3}={k}_{4}={k}_{5}=2.$ ${F}_{10}^{(5)}$ and ${P}_{10}^{(5)}$ are the corresponding fidelity and success probability.

	$|{{\boldsymbol{\Psi }}}_{{\bf{10}}}^{({\boldsymbol{S}}{\boldsymbol{+}})}\rangle$	$|{{\boldsymbol{\Psi }}}_{{\bf{10}}}^{({\boldsymbol{S}}{\boldsymbol{-}})}\rangle$
${F}_{10}^{(5)}$	0.975	0.973
*α*	−0.2	−0.28
${P}_{10}^{(5)}$	0.0008	0.0012
*α*_1_	0	$0.034\cdot exp(i0.94\pi )$
*α*_2_	$1.216\cdot exp(i0.588\pi )$	$1.585\cdot exp(-i0.686\pi )$
*α*_3_	$0.738\cdot exp(-i0.982\pi )$	$0.948\cdot exp(i0.31\pi )$
*α*_4_	$0.99\cdot exp(i0.39\pi )$	$1.401\cdot exp(-i0.021\pi )$
*α*_5_	$1.414\cdot exp(-i0.277\pi )$	$0.295\cdot exp(i0.037\pi )$
*t*_1_	$0.468\cdot exp(i1.116\pi )$	$0.444\cdot exp(i1.321\pi )$
*t*_2_	$0.414\cdot exp(i0.57\pi )$	$0.611\cdot exp(i0.037\pi )$
*t*_3_	$0.506\cdot exp(i0.628\pi )$	$0.702\cdot exp(i1.084\pi )$
*t*_4_	$0.868\cdot exp(i1.668\pi )$	$0.876\cdot exp(i0.087\pi )$
*t*_5_	$0.754\cdot exp(i1.223\pi )$	$0.767\cdot exp(i0.537\pi )$

**Figure 3:**
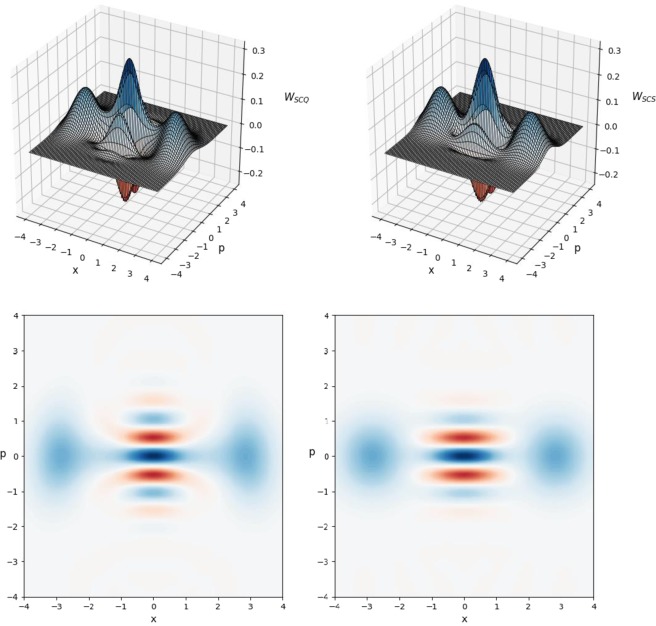
Plot of even Wigner function *W*_*SCQ*_ with $m=3,\,{k}_{0}=4,\,{k}_{1}=\,{k}_{2}={k}_{3}=2$ (left-upper subfigure) and its contour image (left-bottom subfigure) generated in optical scheme in Fig. 2 with parameters taken from Table 3 in comparison with genuine even Wigner function *W*_*SCS*_ with size *β* = 2 (right-upper subfigure) and its contour image (right-bottom subfigure). The fidelity calculated by using Wigner functions of generated and genuine states gives the result ${F}_{10}=0.980140336082$ comparable to that presented in Table 3.

**Figure 4:**
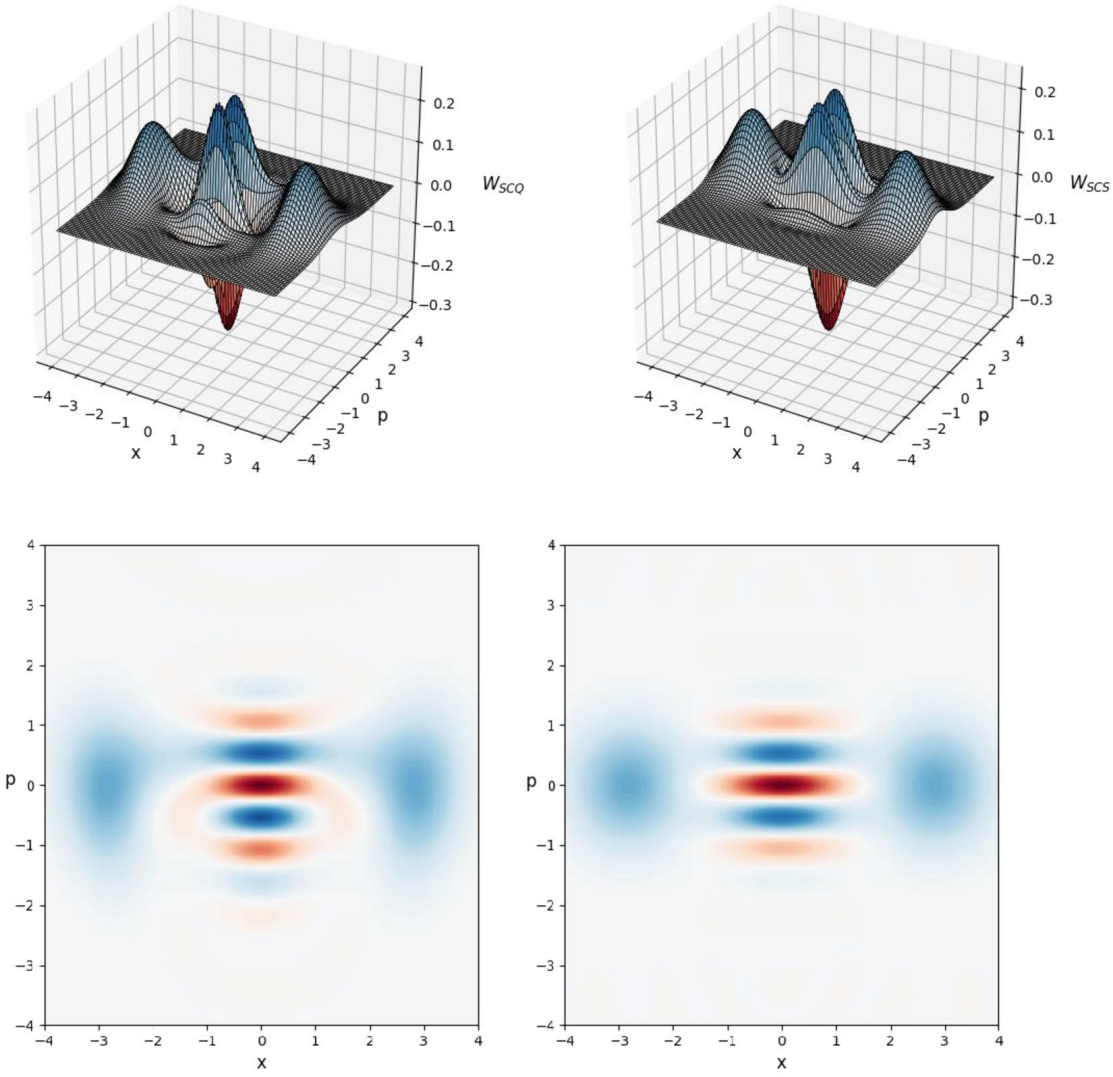
Plot of odd Wigner function *W*_*SCQ*_ with $m=3,\,{k}_{0}=4,\,{k}_{1}=\,{k}_{2}={k}_{3}=2$ (left-upper subfigure) and its contour image (left-bottom subfigure) generated in optical scheme in Fig. 2 with parameters taken from Table 3 in comparison with genuine odd Wigner function *W*_*SCS*_ with size *β* = 2 (right-upper subfigure) and its contour image (right-bottom subfigure). The fidelity calculated by using Wigner functions of generated and genuine states gives the result ${F}_{10}=0.961285449744$ comparable to that presented in Table 3.

As can be seen from Tables 3–5, generation of SCQs with size as large as *β* = 2 is possible in all the three cases with high enough fidelity whose values range from 0.961 up to 0.985. The obtained success probabilities to generate the SCQs are quite small but these are typical for this kind of state generation. Generally speaking, none of the proposed interpretations provides significant advantages over each other. Nevertheless, the data from the Tables reveal the correctness of the proposed scheme which allows us to realize SCQs of large size starting from an original tensor product of Fock states. Since the SCQ generation in the case (i) requires a smaller number of beam splitters, displacement operators and photo-detectors than in the cases (ii) and (iii), this case can be regarded as more effective from an experimental point of view. Finally, to confirm the correctness of the proposed scheme which is rather complicated from a numerical point of view, we use the numerical values of the amplitudes of the superposition in Eq. (34) to construct Wigner functions of the generated (left subfigures) and those of the genuine SCS $|{\beta }_{+}\rangle$ with *β* = 2 (right subfigures). SCQs and compare them with the Wigner functions of the corresponding genuine SCSs. In Fig. 3, we use the numerical data in Table 3 to plot Wigner functions of the SCQ $|{\Psi }_{10}^{(S+)}\rangle$ (left subfigures) and those of the genuine SCS $|{\beta }_{+}\rangle$ with *β* = 2 (right subfigures). The fidelity calculated for the two Wigner functions gives the following value ${F}_{10}=0.980140336082$ which completely coincides with the value of the fidelity presented in Table 3. Also, we show in Fig. 4 Wigner functions of the SCQ $|{\Psi }_{10}^{(S-)}\rangle$ (left subfigures) and the genuine SCS $|{\beta }_{-}\rangle$ with *β* = 2 (right subfigures). Again, the fidelity calculated using the Wigner functions gives the value ${F}_{10}=0.961285449744$ which is the same as that presented in Table 3. Note that the Wigner functions *W*_*SCQ*_ and *W*_*SCS*_ of both the generated SCQ and the genuine SCS exhibit areas of negativity (i.e., areas for which ${W}_{SCQ},\,{W}_{SCS} < 0$), which is a specific feature to ensure nonclassicality of the states of concern. This observation and the full coincidence of the values of fidelities calculated by two different ways allow us to positively judge the relevance of the proposed scheme to generate large-size SCQs from the Fock states.

 As can be seen from the Tables 3–5, the values of experiment parameters (*α*_*i*_, *t*_*i*_) generally take the complex values and, in general, do not allow us to intuitively grasp what caused them and trace the relationship among the parameters. This is partly due to the fact that the parameters used must ensure the alternation of the imaginary unit in superposition terms of the generated states as shown above. It may also be connected with the complex structure of the roots of the polynomial in Eq. (38) that are dependent on amplitudes of ideal SCQs in Eqs (5, 6). Consider it on example of SCQs in 0-representation. Then, we have analytical expressions of purely imaginary roots ${\gamma }_{1}^{(+)}=i\sqrt{2}{\beta }^{-1}\sqrt{3+\sqrt{3}}$, ${\gamma }_{2}^{(+)}=-\,i\sqrt{2}{\beta }^{-1}\sqrt{3+\sqrt{3}}$, ${\gamma }_{3}^{(+)}=i\sqrt{2}{\beta }^{-1}\sqrt{3-\sqrt{3}}$, ${\gamma }_{4}^{(+)}=-\,i\sqrt{2}{\beta }^{-1}\sqrt{3-\sqrt{3}}$ for even SCQ with $n=4$ and complex roots ${\gamma }_{1}^{(-)}=0$, ${\gamma }_{2}^{(-)}={\beta }^{-1}\sqrt{-10+i2\sqrt{5}}$, ${\gamma }_{3}^{(-)}=\,-\,{\beta }^{-1}\sqrt{-10+i2\sqrt{5}}$, ${\gamma }_{4}^{(-)}={\beta }^{-1}\sqrt{-10-i2\sqrt{5}}$, ${\gamma }_{5}^{(-)}=\,-\,{\beta }^{-1}\sqrt{-10-i2\sqrt{5}}$ for odd SCQ with *n* = 5. This must lead to the complex structure of the parameters $({\alpha }_{i},{t}_{i})$ presented in the Tables 3–5 which can be calculated only by numerical simulation.

**Figure 5:**
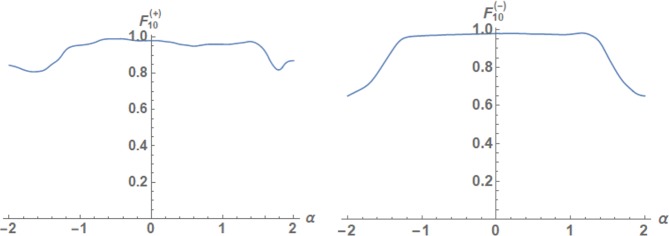
Dependencies of the fidelities ${F}_{10}^{(+)}$ (left-hand side) and ${F}_{10}^{(-)}$ (right-hand side) between displaced qudits generated in the optical scheme in Fig. 2 with *n* = 10 photons in *m* = 5 modes and even/odd SCS of amplitude *β* = 2 on the displaced amplitude *α*.

 We also note the fact that the behavior of the fidelities and probabilities of the generated states in Fig. 2, depending on the variable parameters, is complex and difficult to explain from a logical point of view. For example, we observed through numerical simulation that the maximum fidelity of the output state can be accompanied by a significant decrease (by several orders of magnitude) in the success probability of the target state while the gain in the fidelity can be insignificant (about 1–2 percent). For this reason, we used only the optimal values in Tables 3–5 which provide a sufficiently high fidelity and success probability of the output state. The optical scheme in Fig. 2 allows high variability, which demonstrates the dependence ${F}_{10}^{(+)}$ and ${F}_{10}^{(-)}$ for the case of *m* = 5 on the parameter *α* in Fig. 5. In particular, we present the values of the fidelities, success probabilities and experimental parameters for which they are observed in Table 5 for optimal displacement amplitudes *α* = −0.2 and *α* = −0.28, respectively. The graph of the dependence of the probability of success on *α* has a more complex form with large oscillation amplitudes.

## Discussion

We have considered novel ways to generate displaced qudits, called Schrodinger cat qudits, which may approximate Schrodinger cat states of large size with high fidelity. First, we developed a theory of *α*-representation of the Schrodinger cat states (Eqs (5, 6)), where the quantity *α* takes pure imaginary values. The amplitudes of even and odd Schrodinger cat states are shifted relative to each other by *π*/2. Therefore, the division of the states onto even and odd can be made only in number states base (0-representation). These states have both even and odd amplitudes in any other Hilbert space defined by the displacement amplitude *α*. Schrodinger cat qudits are determined in an (*n* + 1)-dimensional Hilbert space with displaced base elements in Eqs (10, 11) shifted by quantity *α* on phase plane regarding the number states. Schrodinger cat qudits give maximal fidelity with exact Schrodinger cat states for any values of the displacement amplitude *α*. The more the number of terms *n* in the displaced qudit we take, the higher fidelity we can approximate the Schrodinger cat states of large size (see Supplementary Figures 1–4). It is interesting to note that even and odd Schrodinger cat qudits have maximum fidelity in 0-representation for *n* being even and odd, respectively.

Then, we propose possible methods of generating Schrodinger cat qudits. One method is based on a two-mode entangled state in Eq. (15) containing *n* photons in total. The amplitudes of this state follow from Eqs (22, 23) and depend on both Schrodinger cat states amplitudes in Eqs (5, 6) and decomposition coefficients. The generation of even/odd Schrodinger cat qudits in optical scheme in Fig. 1 can be performed with a fairly high probability of success (see Supplementary Figures 5–8). It is shown^50^ that the two-mode entangled *n*-photon state can be realized with the help of two SPDCs and a system of the beam splitters with parameters (Eqs (32, 33)) determined by the roots of the equation in Eq. (29). After such an entangled state in Eq. (15) is produced offline like quantum channel in^5^, either even or odd Schrodinger cat qudits can be generated using the amplitude displacement both in the main and auxiliary modes with the subsequent registration of a specific measurement outcome in number state basis. Potentially, this scheme allows one to realize Schrodinger cat qudits with a size greater than or equal to two (*β* ≥ 2) with an increase in the number *n* of photons used. Despite the simplicity of implementation of the conditional Schrodinger cat qudit generation, this scheme requires quantum channel^5^, realization of which may require great efforts. In order to seek for more possibilities of implementing large-size Schrodinger cat qudits, we proposed another scheme without using the initial two-mode entangled *n*-photon state. Instead, *m* + 1 (*m* ≥ 1) photon number states are used as the input states. With the help of photo-detectors and linear optics devices with properly chosen parameters and arranged as in Fig. 2, large-size Schrodinger cat qudits with high fidelity with the desired Schrodinger cat states can be obtained if no detectors click. The relevance of the method of generation of the desired Schrodinger cat states from photon Fock states is confirmed by means of Wigner functions.

 The main advantage of our method over other approaches (photon number subtraction^26,41–43^ and the breeding protocol^51^) is the variety of strategies that can be implemented within the framework of the approach. In particular, the plots in Fig. 5 confirm the fact. These strategies can be aimed both at increasing the success probability (for example, reducing the number of the beam splitters in Fig. 2), and at increasing the size (*β* > 2) of the generated SCSs as accurate as possible by increasing the number of the beam splitters. The optical scheme in Fig. 2 can allow different inputs in the main 0 mode (for example, superposition states, low-amplitude SCSs to increase their size, and so on) and various states in auxiliary modes (for example, entangled states and even a two-mode squeezed vacuum state). Note that, in general, the optical scheme in Fig. 1 uses a completely accessible resource: two-mode squeezed vacuum state from which ideal SCQs in Eqs (10, 11) are produced. The scheme for generating an auxiliary two-mode entangled state in Eq. (15) is a rather nontrivial task. But it is quite possible in the future to simplify this multi-stage scheme in Fig. 1 to directly use radiation of pair of squeezers for generation of needed SCQs. The search for the best strategy regarding the size of the SCSs, the success probability and the resources that should be spent is the subject of the separate study.

 In general, the optical scheme in Fig. 2 can also be quite robust against photon loss, since the high fidelity of the output states is observed in a wide range of the parameters used. A more accurate answer concerning the effect of loss or/and decoherence can be obtained in a separate study.

## Supplementary information

supplementary information

## Data Availability

The data that support the findings of this study are available from one of the corresponding authors (S.A.P.) upon reasonable request.
