# Supplementary material for: Efficient production of large-size optical Schrödinger cat states
Source: Sci Rep. 2019 Oct 4;9:14301. doi: 10.1038/s41598-019-50703-1 (PMC6778084; doi:10.1038/s41598-019-50703-1)
Supplement: Supplementary file 1 — supplementary information [file 41598_2019_50703_MOESM1_ESM.docx]

**SUPPLEMENTARY NOTE 1: THE** $\boldsymbol{\alpha}$**-REPRESENTATION OF THE SCS**

The displaced number states^1^ are defined through a unitary operator called the displacement operator $D\left( \alpha\right)=exp\left( \alpha a^{+}-\alpha^{*}a \right)$ acting on a Fock state $\left. |n \right\rangle$ as

$\left. |n,\alpha\right\rangle=D\left( \alpha\right)\left. |n \right\rangle$, (S1)

where $\alpha$ is a complex number in general and $a$ $(a^{+})$ the bosonic annihilation (creation) operator^2^. Set of the displaced number states of light

$\left\{ \left. |n,\alpha\right\rangle,n=0,1,2,\ldots,\infty\right\}$ (S2)

is complete for a given $\alpha$. Therefore, any state can be decomposed in terms of the displaced number states with respective coefficients. We name such decomposition $\alpha$-representation. In particular, for $\alpha=0$, the $0$-representation is nothing else but the decomposition in terms of the number states. So, the $0$-representation of the even and odd SCSs in Eqs. (1, 2) of Main Material can be written as column-vectors with infinite number of elements as

$\left. |\beta_{+} \right\rangle=\left[ \begin{matrix} a_{0}^{\left( + \right)} \\ a_{1}^{\left( + \right)} \\ \begin{matrix} a_{2}^{\left( + \right)} \\ a_{3}^{\left( + \right)} \\ \begin{matrix} a_{4}^{\left( + \right)} \\ a_{5}^{\left( + \right)} \\ \begin{matrix} a_{6}^{\left( + \right)} \\ a_{7}^{\left( + \right)} \\ \vdots\end{matrix} \end{matrix} \end{matrix} \end{matrix} \right]=G_{+}\left[ \begin{matrix} 1 \\ 0 \\ \begin{matrix} {\beta^{2}}/\sqrt{2!} \\ 0 \\ \begin{matrix} {\beta^{4}}/\sqrt{4!} \\ 0 \\ \begin{matrix} {\beta^{6}}/\sqrt{6!} \\ 0 \\ \vdots\end{matrix} \end{matrix} \end{matrix} \end{matrix} \right]$, (S3)

$\left. |\beta_{-} \right\rangle=\left[ \begin{matrix} a_{0}^{\left( - \right)} \\ a_{1}^{\left( - \right)} \\ \begin{matrix} a_{2}^{\left( - \right)} \\ a_{3}^{\left( - \right)} \\ \begin{matrix} a_{4}^{\left( - \right)} \\ a_{5}^{\left( - \right)} \\ \begin{matrix} a_{6}^{\left( - \right)} \\ a_{7}^{\left( - \right)} \\ \vdots\end{matrix} \end{matrix} \end{matrix} \end{matrix} \right]=G_{-}\left[ \begin{matrix} 0 \\ \beta\\ \begin{matrix} 0 \\ {\beta^{3}}/\sqrt{3!} \\ \begin{matrix} 0 \\ {\beta^{5}}/\sqrt{5!} \\ \begin{matrix} 0 \\ {\beta^{7}}/\sqrt{7!} \\ \vdots\end{matrix} \end{matrix} \end{matrix} \end{matrix} \right]$, (S4)

where $G_{\pm}=2N_{\pm}\left( \beta\right)exp\left( {-\left| \beta\right|^{2}}/2 \right)$ are the normalization factors. Note also that $\left. |\beta_{+} \right\rangle$ contains only amplitudes proportional to $\beta^{2k}$, while $\left. |\beta_{-} \right\rangle$ is realized with amplitudes proportional to $\beta^{2k+1}.$ The $0$-representation of the displaced number state itself is

$\left. \left| k,\alpha\right. \right\rangle=F(\alpha)\sum_{n=0}^{\infty} c_{kn}\left( \alpha\right)\left. \left| n \right. \right\rangle$, (S5)

Where $F(\alpha)=exp\left( {-\left| \alpha\right|^{2}}/2 \right)$ is the normalization factor and the matrix elements $c_{kn}\left( \alpha\right)$ are the decomposition coefficients of the displaced number state $\left. \left| k,\alpha\right. \right\rangle$ over the number states $\left. |n \right\rangle$ (see Ref. 3), which satisfy the condition $F^{2}(\alpha)\sum_{m=0}^{\infty} \left| c_{kn}\left( \alpha\right) \right|^{2}=1$ because $\left. \left| k,\alpha\right. \right\rangle$ is normalized to 1. These coefficients are the matrix elements of the transformation matrix $U$ which can be built from elements $c_{kn}\left( \alpha\right)$ in Eq. (S5) if $k$ and $n$ change from $0$ up to $\infty$ (see Ref. 3). To get rid of the tedious calculations associated with the multiplication of the unitary infinite transformation matrix by the column vector^3^, we directly obtain the amplitudes of the even/odd SCS in arbitrary $\alpha$-representation. Let us do the mathematical calculations for amplitudes of even SCS in infinite Hilbert space of the displaced number states $\left. |k,\alpha\right\rangle$. The amplitude $a_{k}^{(+)}$ of even SCS in $\alpha$-representation can be calculated as

$a_{k}^{\left( + \right)}=\left\langle k, \alpha| even \right\rangle=N_{+}\left( \left\langle k, \alpha| -\beta\right\rangle+\left\langle k, \alpha| \beta\right\rangle\right)=$

$N_{+}\left( \left\langle k | D\left( -\alpha\right)D\left( -\beta\right) | 0 \right\rangle+\left\langle k | D\left( -\alpha\right)D\left( \beta\right) | 0 \right\rangle\right)$, (S6)

due to completeness of the base displaced number states. Using the operator theorem^2^,

$D\left( \alpha\right)D\left( \beta\right)=D\left( \alpha+\beta\right)exp\left( \frac{\alpha\beta^{*}-\alpha^{*}\beta}{2} \right)=D\left( \alpha+\beta\right)exp\left( iIm\left( \alpha\beta^{*} \right) \right)$, (S7)

applied to the displacement operators, we have from (S6)

$a_{k}^{\left( + \right)}=N_{+}\left( \left\langle k | -\alpha-\beta\right\rangle exp\left( \frac{\alpha\beta^{*}-\alpha^{*}\beta}{2} \right)+\left\langle k | -\alpha+\beta\right\rangle exp\left( \frac{-\alpha\beta^{*}+\alpha^{*}\beta}{2} \right) \right)=$

$N_{+}\left( exp\left( -\frac{\left| -\alpha-\beta\right|^{2}}{2} \right)exp\left( \frac{\alpha\beta^{*}-\alpha^{*}\beta}{2} \right)\frac{\left( -\alpha-\beta\right)^{k}}{\sqrt{k!}}+exp\left( -\frac{\left| -\alpha+\beta\right|^{2}}{2} \right)exp\left( \frac{-\alpha\beta^{*}+\alpha^{*}\beta}{2} \right)\frac{\left( -\alpha+\beta\right)^{k}}{\sqrt{k!}} \right)$. (S8)

Finally, we need to group the phase factors

$\exp\left( \frac{1}{2}\left( -\left( -\alpha-\beta\right)\left( -\alpha^{*}-\beta^{*} \right)+\alpha\beta^{*}-\alpha^{*}\beta\right) \right) =$

$\exp\left( \frac{1}{2}\left( -\alpha\alpha^{*}-\alpha\beta^{*}-\beta\alpha^{*}-\beta\beta^{*}+\alpha\beta^{*}-\alpha^{*}\beta\right) \right) =$

$\exp\left( -\frac{1}{2}\mathbb{a}^{2}-\alpha^{*}\beta\right)$, (S9)

in the first term of (S8) and

$\exp\left( \frac{1}{2}\left( -\left( -\alpha+\beta\right)\left( -\alpha^{*}+\beta^{*} \right)-\alpha\beta^{*}+\alpha^{*}\beta\right) \right) =$

$\exp\left( \frac{1}{2}\left( -\alpha\alpha^{*}+\alpha\beta^{*}+\beta\alpha^{*}-\beta\beta^{*}-\alpha\beta^{*}+\alpha^{*}\beta\right) \right) =$

$\exp\left( -\frac{1}{2}\mathbb{a}^{2}+\alpha^{*}\beta\right)$, (S10)

in the second term of (S8). Inserting all the phase factors into (S8), we obtain

$a_{k}^{\left( + \right)}=\frac{N_{+}}{\sqrt{k!}}\exp\left( -\frac{1}{2}\mathbb{a}^{2} \right)\left( \left( -\alpha-\beta\right)^{k}exp\left( -\alpha^{*}\beta\right)+\left( -\alpha+\beta\right)^{k}exp\left( \alpha^{*}\beta\right) \right)$. (S11)

Similarly, the amplitudes $a_{k}^{\left( - \right)}$ of odd SCS can be derived from relation

$a_{k}^{\left( - \right)}=\left\langle k, \alpha| odd \right\rangle$. (S12)

Now, let us derive the Eqs. (5, 6) of Main Material. For that purpose we turn to the polar coordinates, given that $\beta>0$ and the displacement amplitude $i\alpha$ is pure imaginary. Then, we have $\left( -\alpha-\beta\right)^{k}=\mathbb{a}^{k}exp\left( ik\varphi\right)exp\left( ik\pi\right)$ and $\left( -\alpha+\beta\right)^{k}=\mathbb{a}^{k}exp\left( -ik\varphi\right)$, where the angle on phase space is determined in Main Material. Substituting the expressions into formulas for $a_{k}^{\left( \pm\right)}$, one obtains

$a_{k}^{\left( + \right)}=\frac{N_{+}}{\sqrt{k!}}\exp\left( -\frac{\mathbb{a}^{2}}{2} \right)\mathbb{a}^{k}\left( exp\left( i\alpha\beta+ik\varphi+ik\pi\right)+exp\left( -i\alpha\beta-ik\varphi\right) \right)$=

$\frac{N_{+}}{\sqrt{k!}}\exp\left( -\frac{\mathbb{a}^{2}}{2} \right)\mathbb{a}^{k}exp\left( {ik\pi}/2 \right)\left( exp\left( i\alpha\beta+ik\varphi+{ik\pi}/2 \right)+exp\left( -i\alpha\beta-ik\varphi-{ik\pi}/2 \right) \right)=$

$\frac{N_{+}}{\sqrt{k!}}2\left( i\mathbb{a} \right)^{k}\exp\left( -\frac{\mathbb{a}^{2}}{2} \right)cos\left( \alpha\beta+k\left( \varphi+\pi/2 \right) \right)$, (S13)

$a_{k}^{\left( - \right)}=\frac{N_{-}}{\sqrt{k!}}\exp\left( -\frac{\mathbb{a}^{2}}{2} \right)\mathbb{a}^{k}\left( exp\left( i\alpha\beta+ik\varphi+ik\pi\right)-exp\left( -i\alpha\beta-ik\varphi\right) \right)$=

$\frac{N_{-}}{\sqrt{k!}}\exp\left( -\frac{\mathbb{a}^{2}}{2} \right)\mathbb{a}^{k}exp\left( {ik\pi}/2 \right)\left( exp\left( i\alpha\beta+ik\varphi+{ik\pi}/2 \right)-exp\left( -i\alpha\beta-ik\varphi-{ik\pi}/2 \right) \right)=$

$i\frac{N_{+}}{\sqrt{k!}}2\left( i\mathbb{a} \right)^{k}\exp\left( -\frac{\mathbb{a}^{2}}{2} \right)sin\left( \alpha\beta+k\left( \varphi+\pi/2 \right) \right)$. (S14)

We neglected the overall phase factor $i$ in Eq. (S14) that does not affect anything and get the final expressions for the amplitudes of the SCS in polar coordinates as in Eqs. (5, 6) of Main Material. If we pull the common factor $N_{\pm}exp\left( {-\mathbb{a}^{2}}/2 \right)$ out of the bracket, we get the superpositions as in Eqs. (3, 4) of Main Material whose coefficients $a_{k}^{\left( + \right)}$ are now determined by the formulas in Eqs. (5, 6) of Main Material.

Three-dimensional plots of $F_{n}^{\left( S+ \right)}$ and $F_{n}^{\left( S- \right)}$ in Eq. (14) of Main Material in dependency on $\alpha$ and $\beta$ are shown in Supplementary Figures 1 and 2, respectively, where $n$ varies from $2$ up to $9$. A general rule is observed. If the value of $n$ increases, then the values of the fidelities $F_{n}^{\left( S\pm\right)}$ increase too and approaches $1$ starting from some large enough value of $n$ (say, $n\geq9$). The range of the values of $\alpha$ and $\beta$, in which high fidelities are achieved, is also increased. Visually, already with $n=9$ the SCQs very well simulate both even (Supplementary Figure 1) and odd (Supplementary Figure 2) SCSs with the size as large as up to $\beta=2,$ within a quite wide range of the displacement amplitudes from $\alpha=-2$ up to $\alpha=2$. Moreover, the range of values of the displacement amplitude $\alpha,$ within which high fidelities $F_{n}^{\left( S\pm\right)}$ result, is getting wider and wider for increasing $n$. The oscillatory structure of the fidelities in the plots is caused by the ${cos}/{sin}$ dependence of the coefficients $a_{k}^{\left( \pm\right)}$ in Eqs. (5, 6) of Main Material. The coefficients of the even/odd optical SCSs are shifted relative to each other by $\pi/2$ (cosine function transforms to sine with change of the phase $\varphi\to\varphi+\pi/2$). This means that when the fidelity $F_{n}^{\left( S+ \right)}$ attains a local maximal value, the fidelity $F_{n}^{\left( S- \right)}$ takes a local minimum one (i.e., there is a $\pi/2$ phase-shift) under coincidental values of the parameters $\alpha,$ $\beta,$ and vice versa, regardless of $n$.

We also numerically found the maximum values of the fidelities $F_{n,max}^{\left( S+ \right)}$ (top-left) and $F_{n,max}^{\left( S- \right)}$ (top-right) as a function of $\beta$ for different values of $n$ in Supplementary Figure 3. Maximum values of the fidelities $F_{n,max}^{\left( S+ \right)}$ and $F_{n,max}^{\left( S- \right)}$ follow from Supplementary Figures 1 and 2 and are determined when the displacement amplitude $\alpha$ changes with a fixed value $\beta$ of the cat’s size. It is interesting to note that the maximum values of the fidelity $F_{n,max}^{\left( S+ \right)}$ are observed when $n=2, 4, 6, 8$ (i.e., $n$ is even) in the case of $\alpha=0;$that is, when the SCQ is defined in Hilbert space with base number states ($0$-representation), while the maximum values of the fidelity are observed for odd values $n=3, 5, 7, 9$ in the case of $\alpha\neq0$ (bottom-left subfigure in Supplementary Figure 3). Contrary behaviors are found for the fidelities $F_{n,max}^{\left( S- \right)}$. The maximum value of $F_{n,max}^{\left( S- \right)}$ is observed for $\alpha=0$ in the case of $n=3, 5, 7, 9$ but for $\alpha\neq0$ in the case of $n=2, 4, 6, 8$ (bottom-right subfigure in Supplementary Figure 3). Summarizing the data from Supplementary Figures 1 to 3, we list the numerical values of the size $\beta$ of the SCS and the corresponding displacement amplitude $i\alpha$ in Supplementary Table 1 for which both the fidelities $F_{n}^{\left( S+ \right)}$ and $F_{n}^{\left( S- \right)}$ take values greater than 0.99 $\left( F_{n}^{\left( S+ \right)}>0.99,F_{n}^{\left( S- \right)}>0.99 \right)$ for each value of $n$. A further increase in the size $\beta$ leads to the fact that the fidelities take values smaller than $0.99$ $\left( F_{n}^{\left( S+ \right)}<0.99, F_{n}^{\left( S- \right)}<0.99 \right)$ for any value of $\alpha$.

|  | $F_{n}^{\left( S+ \right)}\left( \beta\right)>0.99$ | | $F_{n}^{\left( S- \right)}\left( \beta\right)>0.99$ | |
| --- | --- | --- | --- | --- |
| $n$ | $\alpha$ | $\beta$ | $\alpha$ | $\beta$ |
| $2$ | $0$ | $0.8615$ | $\pm0.3409$ | $0.7209$ |
| $3$ | $\pm0.328$ | $1.0304$ | $0$ | $1.044$ |
| $4$ | $0$ | $1.2724$ | $\pm0.301$ | $1.2267$ |
| $5$ | $\pm0.2824$ | $1.4361$ | $0$ | $1.4574$ |
| $6$ | $0$ | $1.6405$ | $\pm0.266$ | $1.6184$ |
| $7$ | $\pm0.2523$ | $1.7933$ | $0$ | $1.8098$ |
| $8$ | $0$ | $1.9715$ | $\pm0.2404$ | $1.9571$ |
| $9$ | $\pm0.2301$ | $2.1131$ | $0$ | $2.1252$ |

**Supplementary Table 1.** Maximum values of $\beta$ which guarantee the fidelities exceeding $0.99$ with the appropriate values of the displacement amplitude $i\alpha$. An increase in the size $\beta$ decreases the fidelities below $0.99$ $\left( F_{n}^{\left( S+ \right)}<0.99, F_{n}^{\left( S- \right)}<0.99 \right)$ for any value of the displacement amplitude $\alpha$. The displacement amplitudes $\pm\alpha$ are used due to symmetry in Supplementary Figs. 1 and 2.

As mentioned above, the original SCSs $\left. |\beta_{+} \right\rangle$ and $\left. |\beta_{-} \right\rangle$are exactly orthogonal to each other. Then, it is interesting to see to what extent the SCQs $\left. |\Psi_{n}^{\left( S+ \right)} \right\rangle$ and $\left. |\Psi_{n}^{\left( S- \right)} \right\rangle$ are orthogonal to each other. To measure their orthogonality we plot in Supplementary Figure 4 their scalar product

${SP}_{n}=\left\langle\Psi_{n}^{\left( S- \right)} | \Psi_{n}^{\left( S+ \right)} \right\rangle=N_{n}^{\left( S+ \right)}N_{n}^{\left( S- \right)}\sum_{k=0}^{n} a_{k}^{\left( - \right)^{*}}a_{k}^{\left( + \right)}$, (S15)

in dependency on the parameters $\alpha$ and $\beta$. We can see from this graph that the SCQs under study become more and more orthogonal to each other as the number $n$ of terms in the superposition is increasing. The magnitude of ${SP}_{n}$ is almost completely zero in the entire range of the parameters $\alpha$ and $\beta$ for $n=9$ which suggests that the even and odd SCQs, $\left. |\Psi_{n}^{\left( S+ \right)} \right\rangle$ and $\left. |\Psi_{n}^{\left( S- \right)} \right\rangle$ can be regarded as orthogonal ones with $n\geq9,$ for which the fidelities are also close enough to $1$, confirming the self-consistency of the approximation.

**SUPPLEMENTARY NOTE 2: SUCCESS PROBABILITIES FOR SCQs BY SCHEME USING TWO-MODE ENTANGLED STATE**

In this note we deal with success probabilities for generation of SCQs by the scheme using the two-mode entangled state in Eq. (15) of the Main Material. Namely, we build the maximum values of the success probabilities that can be obtained for certain values of the auxiliary parameter $\alpha^{'}$ in dependence on $\alpha$ and $\beta$. Maximal success probabilities $P_{n0}^{\left( S+ \right)}$ and $P_{n1}^{\left( S+ \right)}$ are shown in Supplementary Figures 5 and 6, while quantities $P_{n0}^{\left( S- \right)}$ and $P_{n1}^{\left( S- \right)}$ are displayed in Supplementary Figures 7 and 8, respectively. These values also depend on the number of terms in generated superposition $n$ and on the registered number $k$ of photons$.$ The general tendency is that the approximation under consideration here is better for a larger $n$ but the corresponding maximal success probability decreases with increasing $n$.

**SUPPLEMENTARY NOTE 3: DERIVATION OF FORMULA (34) FOR** $\boldsymbol{m=1}$ **AND** $\boldsymbol{m=2}$

First, consider the simplest case with $m=1$ for which there are two modes: mode $0$ and mode $1.$ Let the states incoming to the beam splitter ${BS}_{01},$ which has transmission (reflection) coefficient $t_{1}$ ($r_{1}$), be $\left. |k_{0} \right\rangle_{0}\left. |k_{1} \right\rangle_{1}=$ $\left. |k_{0}k_{1} \right\rangle_{01}$ with $k_{0}, k_{1}\geq0$ being photon numbers. The beam splitter acts on creation operators like this

$a_{0}^{+}\to t_{1}a_{0}^{+}+r_{1}a_{1}^{+}$, (S16)

$a_{1}^{+}\to-r_{1}^{*}a_{0}^{+}+t_{1}^{*}a_{1}^{+}$. (S17)

By virtue of Eqs. (S16) and (S17), after the beam splitter the input states $\left. |k_{0}k_{1} \right\rangle_{01}$ is transformed to

${BS}_{01}\left. |k_{0}k_{1} \right\rangle_{01}=\frac{1}{\sqrt{k_{0}!k_{1}!}}{BS}_{01}\left( {a_{0}^{+}}^{k_{0}}{a_{1}^{+}}^{k_{1}} \right)\left. \left| 00 \right. \right\rangle_{01}=$

$\frac{\left( t_{1}a_{0}^{+}+r_{1}a_{1}^{+} \right)^{k_{0}}\left( -r_{1}^{*}a_{0}^{+}+t_{1}^{*}a_{1}^{+} \right)^{k_{1}}}{\sqrt{k_{0}!k_{1}!}}\left. |00 \right\rangle_{01}.$ (S18)

The action of the displacement operator $D_{1}\left( \alpha_{1} \right)$ on mode $1$ of the state in Eq. (S18) can be written as

$D_{1}\left( \alpha_{1} \right){BS}_{01}\left. |k_{0}k_{1} \right\rangle_{01}=\frac{1}{\sqrt{k_{0}!k_{1}!}}$

$D_{1}\left( \alpha_{1} \right)\left( t_{1}a_{0}^{+}+r_{1}a_{1}^{+} \right)^{k_{0}}{D_{1}^{+}\left( \alpha_{1} \right)D_{1}\left( \alpha_{1} \right)\left( -r_{1}^{*}a_{0}^{+}+t_{1}^{*}a_{1}^{+} \right)}^{k_{1}}D_{1}^{+}\left( \alpha_{1} \right)D_{1}\left( \alpha_{1} \right)\left. \left| 00 \right. \right\rangle_{01}$ (S19)

thanks to the identity $D_{1}^{+}\left( \alpha_{1} \right)D_{1}\left( \alpha_{1} \right)=1.$ Next, using the properties $D_{1}\left( \alpha_{1} \right){a_{1}^{+}D}_{1}^{+}\left( \alpha_{1} \right){=a}_{1}^{+}-\alpha_{1}^{*}$ and $D_{1}\left( \alpha_{1} \right)\left. \left| 0 \right. \right\rangle_{1}=\left. \left| \alpha_{1} \right. \right\rangle_{1}=exp\left( {-\left| \alpha_{1} \right|^{2}}/2 \right)\sum_{l=0}^{\infty} \frac{\alpha_{1}^{l}}{\sqrt{l!}}\left. \left| l \right. \right\rangle_{1}$ we bring Eq. (48) to Eq. (S18) to get

$D_{1}\left( \alpha_{1} \right){BS}_{01}\left. |k_{0}k_{1} \right\rangle_{01}=\frac{\left( t_{1}a_{0}^{+}+r_{1}\left( a_{1}^{+}-\alpha_{1}^{*} \right) \right)^{k_{0}}\left( -r_{1}^{*}a_{0}^{+}+t_{1}^{*}\left( a_{1}^{+}-\alpha_{1}^{*} \right) \right)^{k_{1}}}{\sqrt{k_{0}!k_{1}!}}\left. \left| 0 \right. \right\rangle_{0}$

$\times exp\left( -\frac{\left| \alpha_{1} \right|^{2}}{2} \right)\sum_{l=0}^{\infty} \frac{\alpha_{1}^{l}}{\sqrt{l!}}\left. \left| l \right. \right\rangle_{1}.$ (S20)

We are interested in the situation when neither detectors click (i.e., no photons are registered at all the detectors). In such situation the post-selected state reads (by formally replacing $a_{1}^{+}$ by zero in Eq. (S20))

$\left. |\Gamma_{n}^{\left( 1 \right)} \right\rangle_{0}=\frac{1}{\sqrt{P_{n}^{\left( 1 \right)}}}\frac{\left( t_{1} \right)^{k_{0}}\left( {-r}_{1}^{*} \right)^{k_{1}}\left( a_{0}^{+}-\frac{r_{1}}{t_{1}}\alpha_{1}^{*} \right)^{k_{0}}\left( a_{0}^{+}-\frac{{-t}_{1}^{*}}{r_{1}^{*}}\alpha_{1}^{*} \right)^{k_{1}}}{\sqrt{k_{0}!k_{1}!}}exp\left( -\frac{\left| \alpha_{1} \right|^{2}}{2} \right)\left. \left| 0 \right. \right\rangle_{0}=$

$\frac{1}{\sqrt{P_{n}^{\left( 1 \right)}}}\frac{\left( t_{1} \right)^{k_{0}}\left( -r_{1}^{*} \right)^{k_{1}}}{\sqrt{k_{0}!k_{1}!}}\left[ D_{0}\left( \frac{r_{1}^{*}}{t_{1}^{*}}\alpha_{1} \right)a_{0}^{+}D_{0}^{\dagger}\left( \frac{r_{1}^{*}}{t_{1}^{*}}\alpha_{1} \right) \right]^{k_{0}}\left[ D_{0}\left( \frac{-t_{1}}{r_{1}}\alpha_{1} \right)a_{0}^{+}D_{0}^{\dagger}\left( \frac{-t_{1}}{r_{1}}\alpha_{1} \right) \right]^{k_{1}}$

$\times exp\left( -\frac{\left| \alpha_{1} \right|^{2}}{2} \right)\left. \left| 0 \right. \right\rangle_{0},$ (S21)

where $n=k_{0}+k_{1}$ and

$P_{n}^{\left( 1 \right)}=\frac{1}{k_{0}!k_{1}!}exp\left( -\left| \alpha_{1} \right|^{2} \right)\sum_{k=0}^{n} \left| m_{k}\left( t_{1},r_{1},\alpha_{1} \right) \right|^{2}k!$, (S22)

is the success probability. Here, the amplitudes $m_{k}\left( t_{1},r_{1},\alpha_{1} \right)$ are obtained by expanding the expression $\left( t_{1}a_{0}^{+}-r_{1}\alpha_{1}^{*} \right)^{k_{0}}\left( -r_{1}^{*}a_{0}^{+}-t_{1}^{*}\alpha_{1}^{*} \right)^{k_{1}}=\sum_{k=0}^{n} m_{k}\left( t_{1},r_{1},\alpha_{1} \right)a_{0}^{+k}$ in powers of the creation operator $a_{0}^{+}$. We do not provide analytical expressions for $m_{k}\left( t_{1},r_{1},\alpha_{1} \right)$ because of their complexity of representation. However, these expressions can be directly obtained in numerical simulation. If we define $N_{n}^{(1)}$ and $\beta_{k}^{(1)}$ as in Eqs. (39) and (40) of Main Material, we can rewrite $\left. |\Gamma_{n}^{\left( 1 \right)} \right\rangle_{0}$ in the following form

$\left. |\Gamma_{n}^{\left( 1 \right)} \right\rangle_{0}=N_{n}^{(1)}\prod_{k=1}^{n} D_{0}\left( \beta_{k}^{(1)*} \right)a^{+}D_{0}^{\dagger}\left( \beta_{k}^{(1)*} \right)\left. \left| 0 \right. \right\rangle_{0},$ (S23)

which upon action of $D_{0}\left( i\alpha\right)$ on mode $0$ yields the output state $\left. |\Omega_{n}^{\left( m \right)} \right\rangle_{0}$ of Eq. (34) in Main Material for $m=1.$

Now, consider the case of $m=2$ for which there are three modes: the principal mode $0$ and two auxiliary modes $2$ and $3.$ The input state is $\left. |k_{0}k_{1}k_{2} \right\rangle_{012},$ with photon numbers $k_{0}, k_{1},k_{2}\geq0.$ Two beam splitters with parameters $\left( t_{1},r_{1} \right)$ and $\left( t_{2},r_{2} \right)$ are used to mix modes $0$, $1$ and modes $0$, $2$, respectively,

$\left. {BS}_{01}^{\left( 1 \right)}{BS}_{02}^{\left( 2 \right)}\left| k_{0}k_{1}k_{2} \right. \right\rangle_{012}=\frac{{BS}_{02}^{\left( 2 \right)}{BS}_{01}^{\left( 1 \right)}{a_{0}^{+}}^{k_{0}}{a_{1}^{+}}^{k_{1}}{a_{2}^{+}}^{k_{2}}}{\sqrt{k_{0}!k_{1}!k_{2}!}}\left. \left| 000 \right. \right\rangle_{012}=$

$\frac{\left( t_{1}\left( t_{2}a_{0}^{+}+r_{2}a_{2}^{+} \right)+r_{1}a_{1}^{+} \right)^{k_{0}}\left( -r_{1}^{*}\left( t_{2}a_{0}^{+}+r_{2}a_{2}^{+} \right)+t_{1}^{*}a_{1}^{+} \right)^{k_{1}}\left( -r_{2}^{*}a_{0}^{+}+t_{2}^{*}a_{2}^{+} \right)^{k_{2}}}{\sqrt{k_{0}!k_{1}!k_{2}!}}\left. \left| 000 \right. \right\rangle_{012}$. (S24)

A subsequent unitary operation is associated with two displacement operators $D_{1}\left( \alpha_{1} \right)$ and $D_{2}\left( \alpha_{2} \right)$ that transform the state in Eq. (S24) into

$\left. {D_{1}\left( \alpha_{1} \right)D_{2}\left( \alpha_{2} \right)BS}_{01}^{\left( 1 \right)}{BS}_{02}^{\left( 2 \right)}\left| k_{0}k_{1}k_{2} \right. \right\rangle_{012}=$

$= \frac{1}{\sqrt{k_{0}!k_{1}!k_{2}!}}\cdot\left( t_{1}\left( t_{2}a_{0}^{+}+r_{2}\left( a_{2}^{+}-\alpha_{2}^{*} \right) \right)+r_{1}\left( a_{1}^{+}-\alpha_{1}^{*} \right) \right)^{k_{0}}$

${\times\left( -r_{1}^{*}\left( t_{2}a_{0}^{+}+r_{2}\left( a_{2}^{+}-\alpha_{2}^{*} \right) \right)+t_{1}^{*}\left( a_{1}^{+}-\alpha_{1}^{*} \right) \right)}^{k_{1}}$

${\times\left( -r_{2}^{*}a_{0}^{+}+t_{2}^{*}\left( a_{2}^{+}-\alpha_{2}^{*} \right) \right)}^{k_{2}}\left. \left| 0\alpha_{1}\alpha_{2} \right. \right\rangle_{012}$. (S25)

If we are again interested in generating the conditional state when no clicks are seen in the auxiliary modes (the state in Eq. (S25) is projected onto $\left. |00 \right\rangle_{12}$), then we can formally replace the creation operation $a_{2}^{+}$ by zero in formula (S25) to obtain the state

$\left. |\Gamma_{n}^{\left( 2 \right)} \right\rangle_{0}=\frac{1}{\sqrt{P_{n}^{\left( 2 \right)}}}\frac{\left( t_{1}t_{2} \right)^{k_{0}}\left( -r_{1}^{*}t_{2} \right)^{k_{1}}\left( -r_{2}^{*} \right)^{k_{2}}}{\sqrt{k_{0}!k_{1}!k_{2}!}}\left( a_{0}^{+}-\frac{t_{1}r_{2}\alpha_{2}^{*}+r_{1}\alpha_{1}^{*}}{t_{1}t_{2}} \right)^{k_{0}}\left( a_{0}^{+}-\frac{r_{1}^{*}r_{2}\alpha_{2}^{*}-t_{1}^{*}\alpha_{1}^{*}}{r_{1}^{*}t_{2}} \right)^{k_{1}}$

${\times\left( a_{0}^{+}-\frac{-t_{2}^{*}\alpha_{2}^{*}}{r_{2}^{*}} \right)}^{k_{2}}exp\left( -\frac{\left| \alpha_{1} \right|^{2}+\left| \alpha_{2} \right|^{2}}{2} \right)\left. \left| 0 \right. \right\rangle_{012}=$

$\frac{1}{\sqrt{P_{n}^{\left( 2 \right)}}}\frac{\left( t_{1}t_{2} \right)^{k_{0}}\left( -r_{1}^{*}t_{2} \right)^{k_{1}}\left( {-r}_{2}^{*} \right)^{k_{2}}}{\sqrt{k_{0}!k_{1}!k_{2}!}}\left[ D_{0}\left( \frac{{t_{1}^{*}r}_{2}^{*}\alpha_{2}+r_{1}^{*}\alpha_{1}}{t_{1}^{*}t_{2}^{*}} \right)a_{0}^{+}D_{0}^{\dagger}\left( \frac{{t_{1}^{*}r}_{2}^{*}\alpha_{2}+r_{1}^{*}\alpha_{1}}{t_{1}^{*}t_{2}^{*}} \right) \right]^{k_{0}}$

$\left[ D_{0}\left( \frac{r_{1}r_{2}^{*}\alpha_{2}-t_{1}\alpha_{1}}{r_{1}t_{2}^{*}} \right)a_{0}^{+}D_{0}^{\dagger}\left( \frac{r_{1}r_{2}^{*}\alpha_{2}-t_{1}\alpha_{1}}{r_{1}t_{2}^{*}} \right) \right]^{k_{1}}\left[ D_{0}\left( -\frac{t_{2}\alpha_{2}}{r_{2}} \right)a_{0}^{+}D_{0}^{\dagger}\left( -\frac{t_{2}\alpha_{2}}{r_{2}} \right) \right]^{k_{2}}$,

$\times exp\left( -\frac{\left| \alpha_{1} \right|^{2}+\left| \alpha_{2} \right|^{2}}{2} \right)\left. \left| 0 \right. \right\rangle_{0}$, (S26)

where $n=k_{0}+k_{1}+k_{2}$ and

$P_{n}^{\left( 2 \right)}=\frac{1}{k_{0}!k_{1}!k_{2}!}exp\left( -\left( \left| \alpha_{1} \right|^{2}+\left| \alpha_{2} \right|^{2} \right) \right)\sum_{k=0}^{n} \left| m_{k}\left( t_{1},r_{1},t_{2},r_{2},\alpha_{1},\alpha_{2} \right) \right|^{2}k!$, (S27)

being the success probability. Here, the amplitudes $m_{k}\left( t_{1},r_{1},t_{2},r_{2},\alpha_{1},\alpha_{2} \right)$ follow from the decomposition of operator expression $\left( t_{1}\left( t_{2}a_{0}^{+}-r_{2}\alpha_{2}^{*} \right)-r_{1}\alpha_{1}^{*} \right)^{k_{0}}\left( -r_{1}^{*}\left( t_{2}a_{0}^{+}-r_{2}\alpha_{2}^{*} \right)-t_{1}^{*}\alpha_{1}^{*} \right)^{k_{1}}\left( -r_{2}^{*}a_{0}^{+}-t_{2}^{*}\alpha_{2}^{*} \right)^{k_{2}}=\sum_{k=0}^{n} m_{k}\left( t_{1},r_{1},\alpha_{1} \right)a_{0}^{+k}$ and are not presented due to their complexity. If we define $N_{n}^{(2)}$ and $\beta_{k}^{(2)}$ as in Eqs. (39) and (40) of Main Material, then we can cast $\left. |\Gamma_{n}^{\left( 2 \right)} \right\rangle_{0}$ into the following form

$\left. |\Gamma_{n}^{\left( 2 \right)} \right\rangle_{0}=N_{n}^{(2)}\prod_{k=1}^{n} D_{0}\left( \beta_{k}^{(2)*} \right)a_{0}^{+}D_{0}^{\dagger}\left( \beta_{k}^{(2)*} \right)\left. \left| 0 \right. \right\rangle_{0}$, (S28)

which, upon the action of $D_{0}\left( i\alpha\right)$ on the principal mode $0,$ is nothing else but the output state $\left. |\Omega_{n}^{\left( m \right)} \right\rangle_{0}$ of Eq. (34) of Main Material for $m=2.$

Likewise, the formula (34) of Main Material can be derived analytically for any $m>2$. However, the formulation gets more cumbersome and thus will not be presented explicitly.

**References to supplementary notes**

1. Podoshvedov, S. A. Generation of displaced squeezed superpositions of coherent states. J. Exp. Theor. Phys. **114**, 451-464 (2012).

2. Walls D. F. & Milburn, G. J. Quantum Optics, (Springer-Verlag, Berlin Heidelberg, 1994).

3. Podoshvedov, S. A. Elementary quantum gates in different bases. Quant. Inf. Proc. **15**, 3967-3993 (2016).


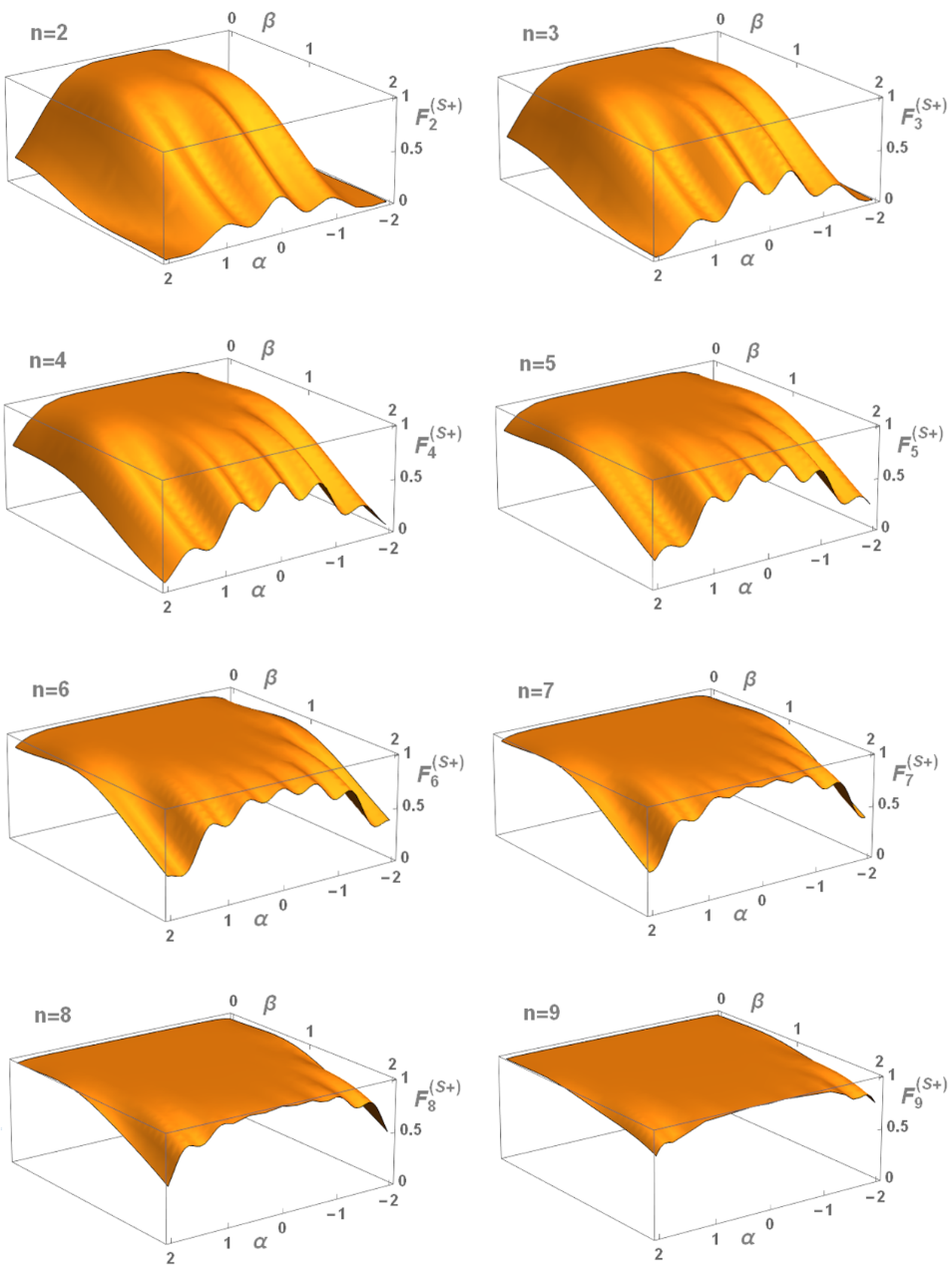


**Supplementary Figure 1.**  Fidelity $F_{n}^{\left( S+ \right)}$ between even SCS, Eq. (1) of Main Material, and its truncated version, Eq. (10) of Main Material, in dependency on its size $\beta$ and displacement amplitude $\alpha$ of the base elements. From top to bottom and from left to right, the SCS dimension grows from $n=2$ up to $n=9$.


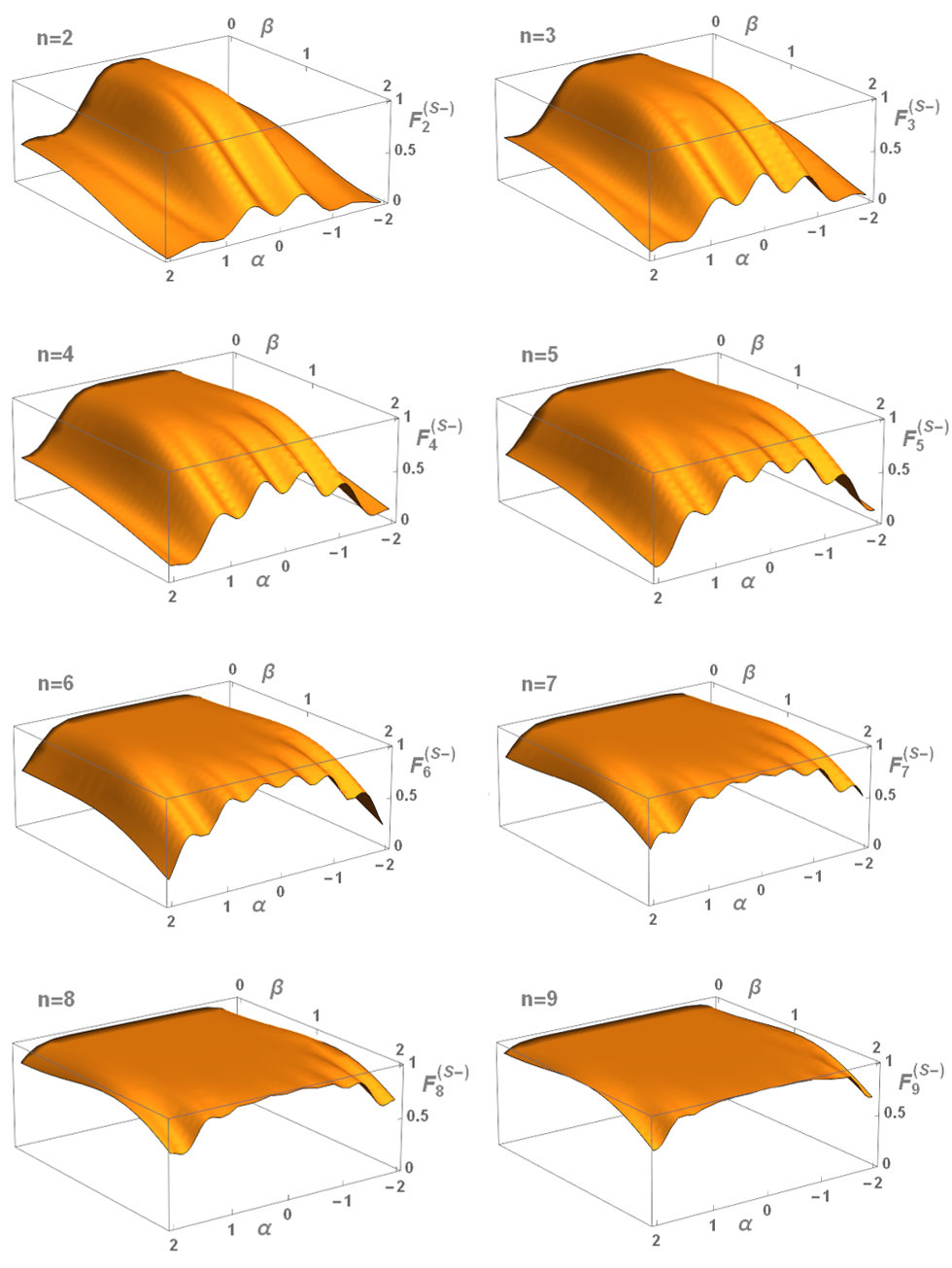


**Supplementary Figure 2.** Fidelity $F_{n}^{\left( S- \right)}$ between even SCS, Eq. (2) of Main Material, and its truncated version, Eq. (11) of Main Material, in dependency on its size $\beta$ and displacement amplitude $\alpha$ of the base elements. From top to bottom and from left to right, the SCS dimension grows from $n=2$ up to $n=9$.


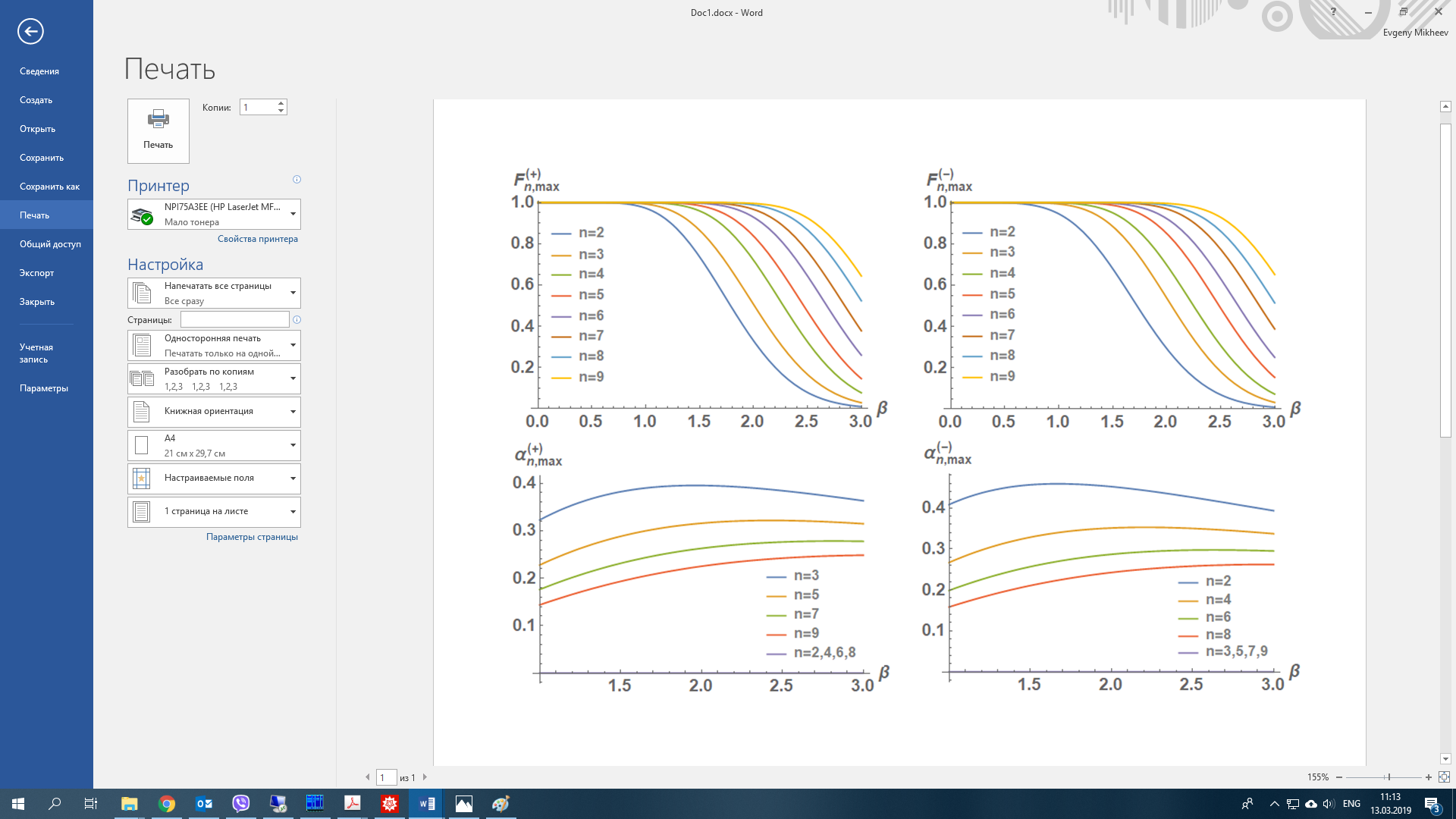


**Supplementary Figure 3.** Maximal fidelities $F_{n,max}^{\left( S+ \right)}$ (top-left) and $F_{n,max}^{\left( S- \right)}$ (top-right) between SCSs, Eqs. (1, 2) of Main Material, and SCQs, Eqs. (10, 11) of Main Material, against its size $\beta$. The displacement amplitude $\alpha_{n,max}^{\left( + \right)}$ (bottom-left) and $\alpha_{n,max}^{\left( - \right)}$ (bottom-right) of the base elements under which the maximal fidelities are observed are shown in dependency on $\beta$.


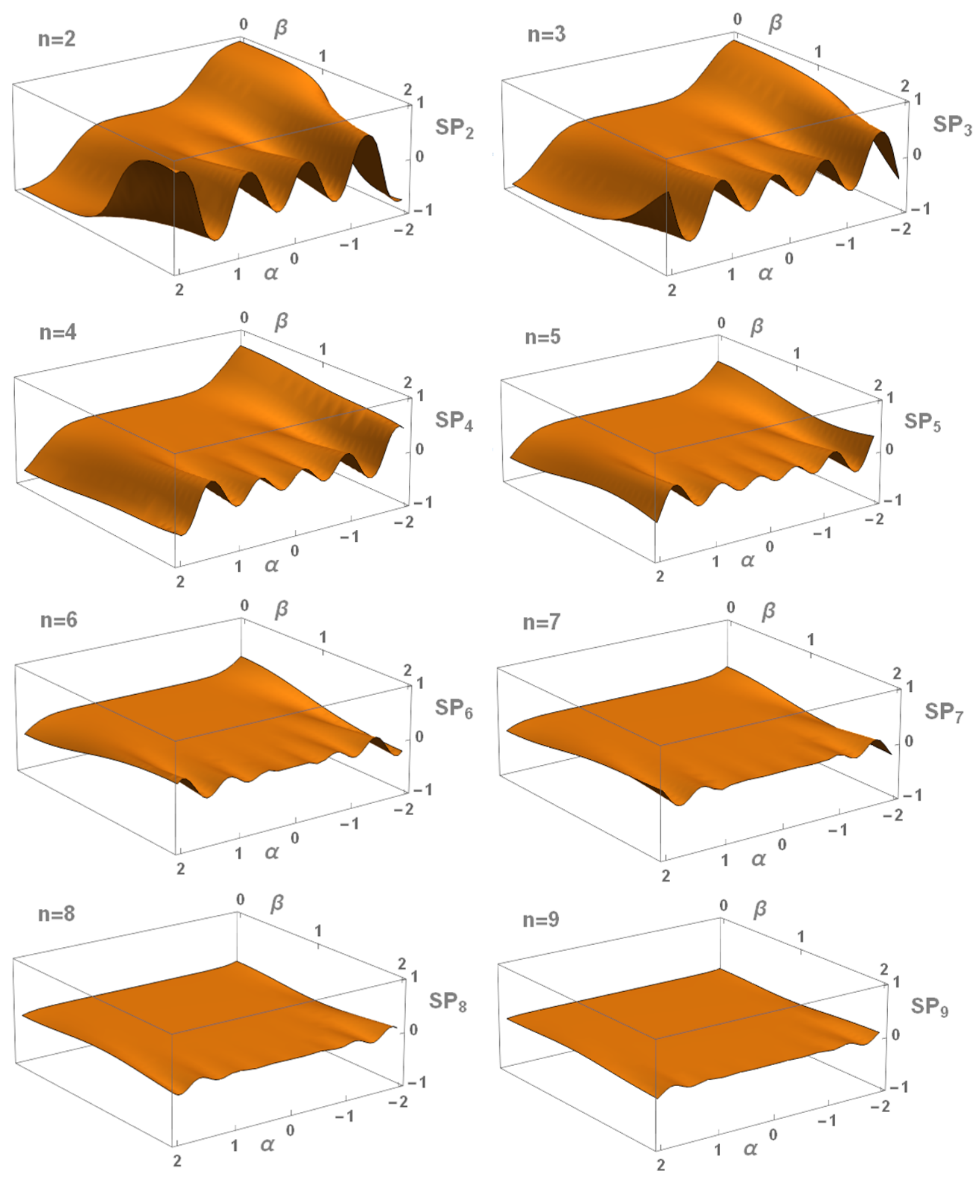


**Supplementary Figure 4.** Absolute value of scalar product ${SP}_{n}$, Eq. (S15), between even and odd truncated versions of SCQs, Eqs. (10, 11) of Main Material, in dependency on size $\beta$ and displacement amplitude $\alpha$ of the base elements. From top to bottom and from left to right, the scalar product $\left| {SP}_{n} \right|$ becomes smaller approaching to zero when $n$ grows from $n=2$ up to $n=9$.


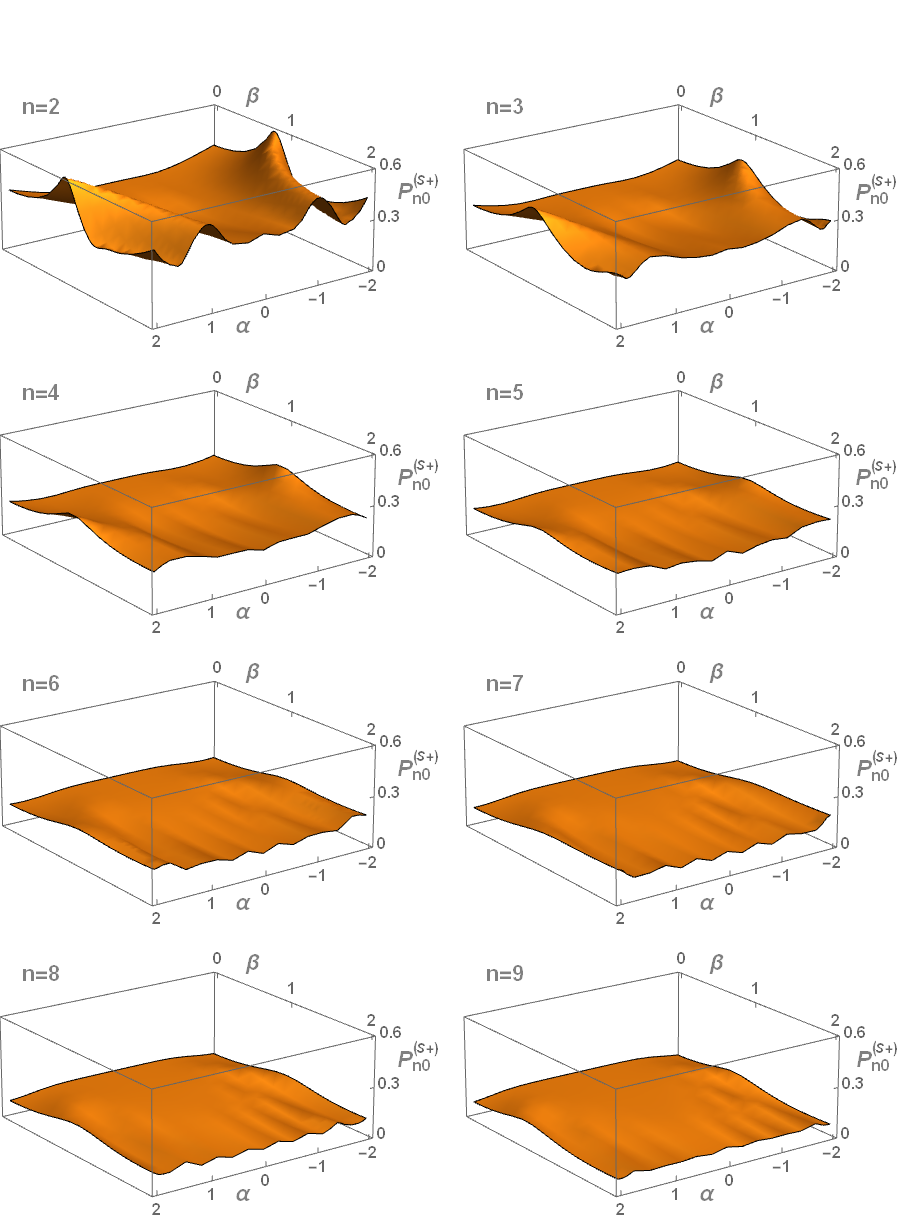


**Supplementary Figure 5.** Three-dimensional plots of maximal success probabilities $P_{n0}^{\left( S+ \right)}$ to generate SCQs, Eqs. (10, 11) of Main Material, in dependency on its size $\beta$ and displacement amplitude $\alpha$. From top to bottom and from left to right, the SCS dimension grows from $n=2$ up to $n=9$.


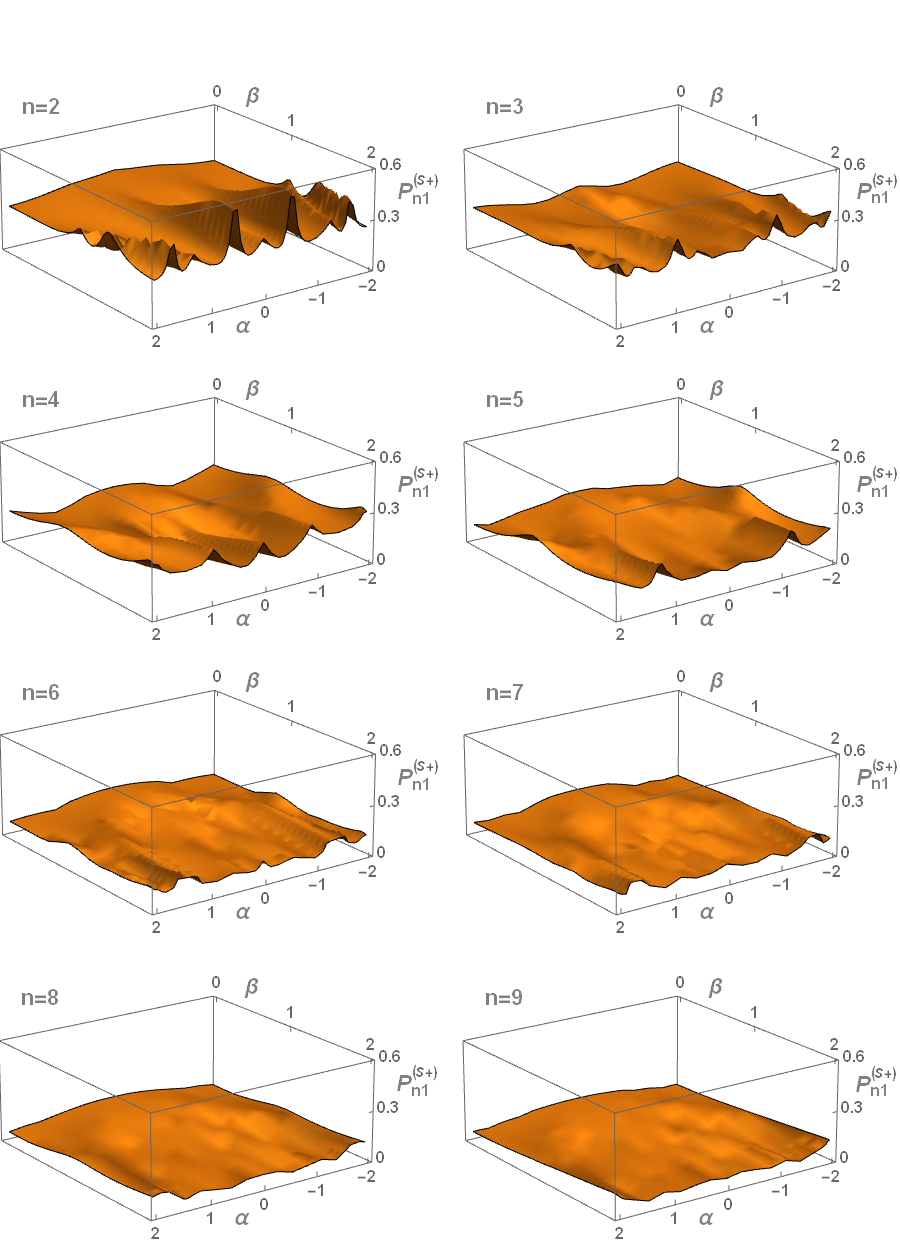


**Supplementary Figure 6.** Three-dimensional plots of maximal success probabilities $P_{n1}^{\left( S+ \right)}$ to generate SCQs, Eqs. (10, 11) of Main Material, in dependency on its size $\beta$ and displacement amplitude $\alpha$. From top to bottom and from left to right, the SCS dimension grows from $n=2$ up to $n=9$.


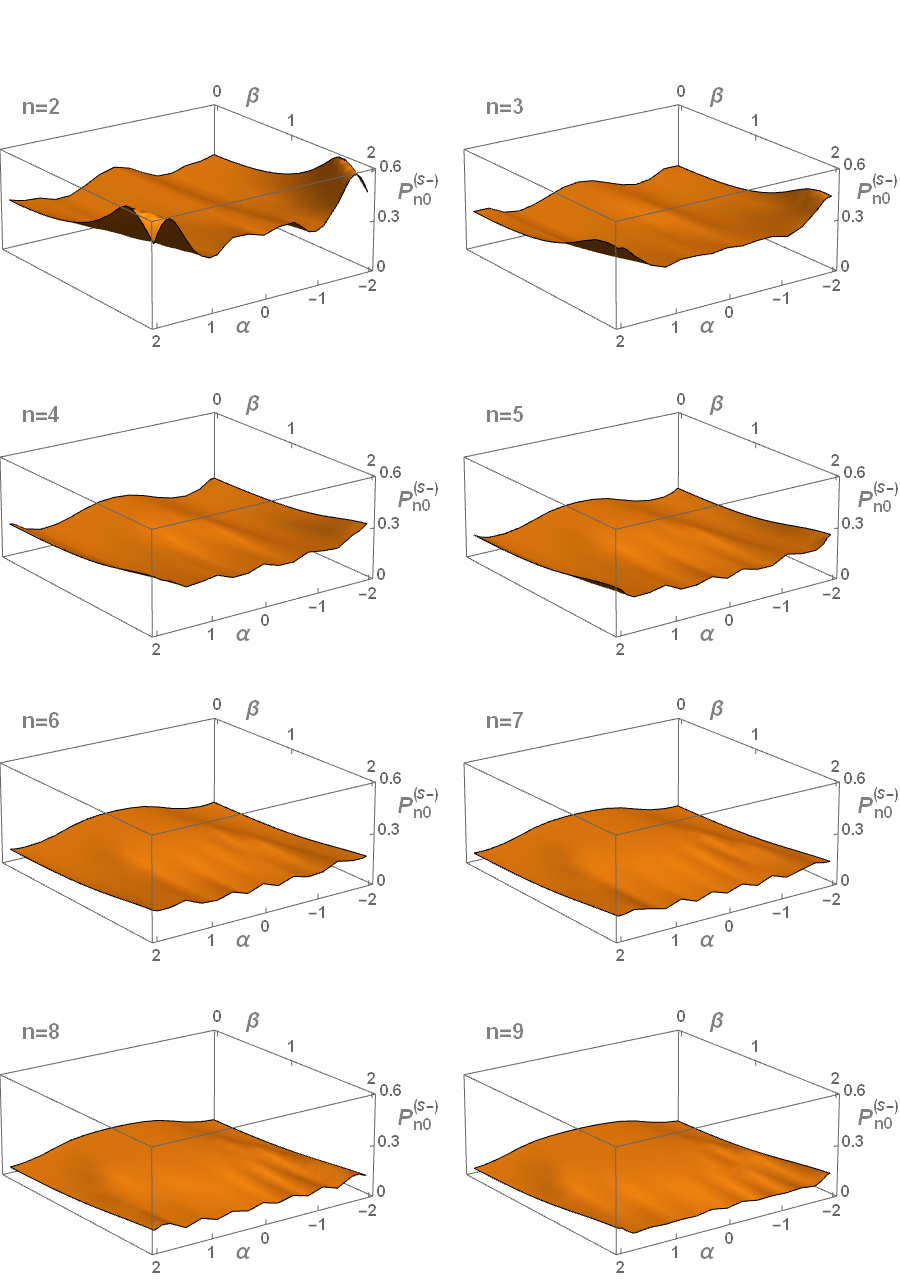


**Supplementary Figure 7.** Three-dimensional plots of maximal success probabilities $P_{n0}^{\left( S- \right)}$ to generate SCQs, Eqs. (10,11) of Main Material, in dependency on its size $\beta$ and displacement amplitude $\alpha$. From top to bottom and from left to right, the SCS dimension grows from $n=2$ up to $n=9$.


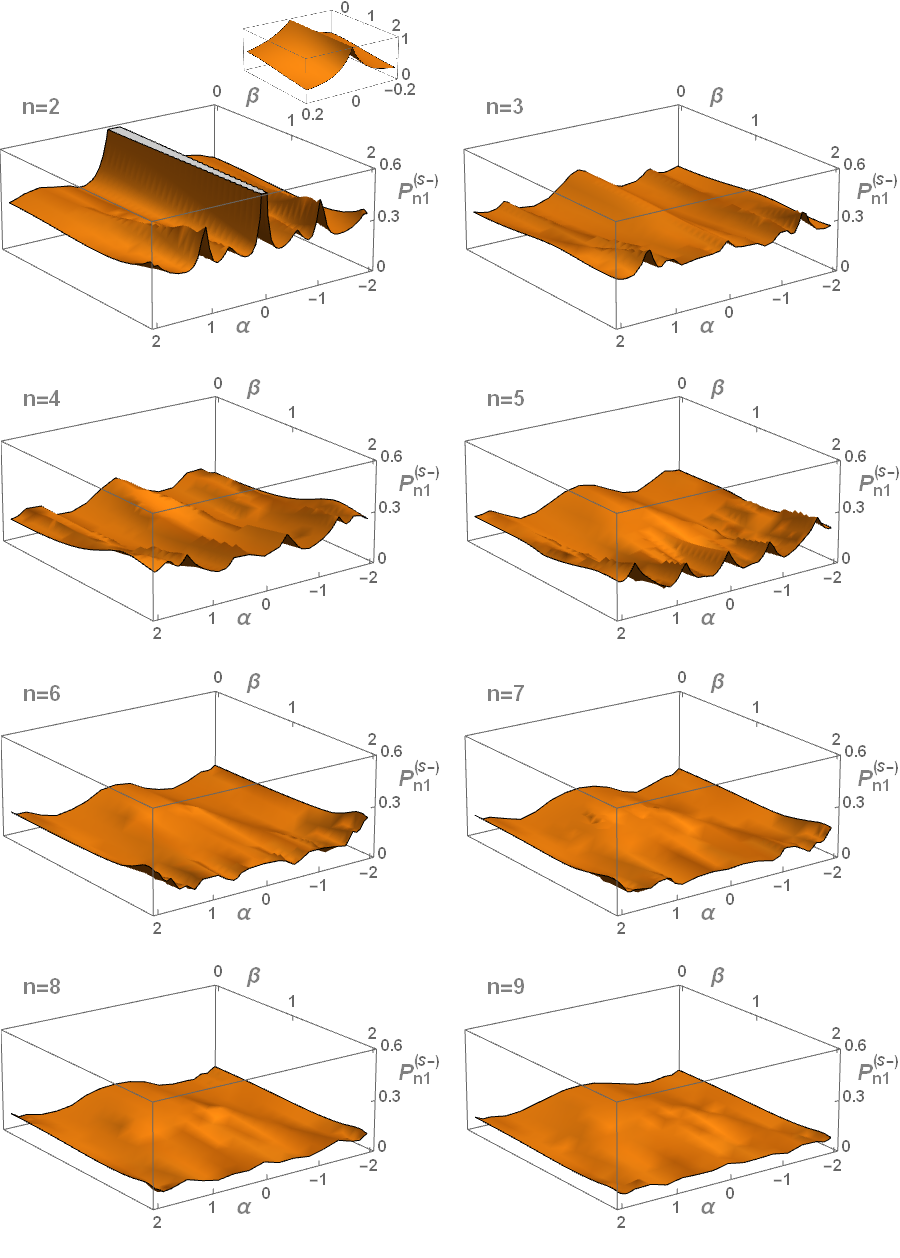


**Supplementary Figure 8.** Three-dimensional plots of maximal success probabilities $P_{n1}^{\left( S- \right)}$ to generate SCQs, Eqs. (10,11) of Main Material, in dependency on its size $\beta$ and displacement amplitude $\alpha$. From top to bottom and from left to right, the SCS dimension grows from $n=2$ up to $n=9$.
